# Active Metamaterials with Tunable Shear Nonreciprocity and Nonlinear Dynamics

**DOI:** 10.1002/advs.74736

**Published:** 2026-03-12

**Authors:** Xin Fang, Miao Yu, Dianlong Yu, Li Cheng

**Affiliations:** ^1^ National Key Laboratory of Equipment State Sensing and Smart Support National University of Defense Technology Changsha Hunan China; ^2^ College of Intelligent Science and Technology National University of Defense Technology Changsha Hunan China; ^3^ Department of Mechanical Engineering Hong Kong Polytechnic University Hong Kong China

## Abstract

Actively and smoothly tunable mechanical metamaterials are in high demand for adaptive, variable‐stiffness structures in smart machines. However, existing designs are largely restricted to tunable transverse deformation, reciprocal response, and linear dynamics. Here, we propose a novel gear‐based design paradigm—using Taiji planar gears and planetary gear assemblies as building blocks—that overcomes these limitations by enabling simultaneous control of translational and torsional stiffnesses, shear nonreciprocity, and programmable nonlinear dynamics. Our metamaterials achieve in situ, continuous tuning of shear stiffness by 30–100×, break reciprocity under positive versus negative loads, and allow the nonreciprocity ratio to be tuned by over 100×. Meta‐resonators constructed from these units showcase an application example exhibit broadly tunable transverse and torsional resonant frequencies. Furthermore, we demonstrate that static nonreciprocity serves as a precise control knob for dynamic nonlinearity—a property traditionally fixed and nearly impossible to tune in conventional materials. Analytical models and analyses elucidate the underlying mechanisms and extendable design freedoms. This work bridges the critical gaps in mechanical metamaterials and dynamics, offering a practical pathway to control both linear and nonlinear structural deformations, elastic waves, and vibrations.

## Graphical abstract

Active gear‐based mechanical metamaterials enable simultaneous control of translational and torsional stiffnesses by 30–100×, break shear reciprocity under positive versus negative loads, broadly tune resonant frequencies, and offer programmable dynamic responses. The tunable process is in situ, continuous, smooth, and completely repeatable, offering practical pathways to control linear and nonlinear structural deformations, elastic waves, and vibrations.

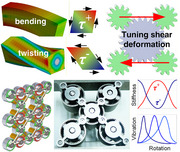

## 1 Introduction

Advanced intelligent systems—from adaptive robotics to morphing aerospace structures— demand components that can dynamically reconfigure their mechanical response [1], wherein compliant structures with *continuously tunable stiffness* are essential for achieving real‐time adaptation to an uncertain environment [2, 3, 4, 5]. For example, vehicle suspensions that modulate stiffness balance ride comfort and speed; robotic limbs adjust rigidity for dexterous manipulation; and aircraft wings morph across flight regimes. Particularly, variable‐stiffness mechanical resonators [6, 7] further underpin critical functions such as sensing, vibration isolation [8], and energy harvesting, and serve as building blocks for locally resonant metamaterials [9, 10] with engineered wave propagation [11, 12] and bandgaps [13, 14, 15, 16].

Great efforts have been made to design tunable structures [17] based on buckling [8, 18, 19, 20], shape‐morphing structures [15] or active materials (e.g., piezoelectrics [9, 21], magnetorheological elastomers [22, 23]). Active tunability can offer excellent environment adaptivity and enables to robustly control the subtle, narrowband, and uncertain phenomena. For example, using the material nonlinearity of soft matter, one can tune the narrowband topological interface states in a soft phononic crystal by using external pre‐stretch force or dielectric effect [24, 25]. However, existing approaches typically offer only abrupt (non‐continuous) stiffness jumps or a limited tunable range. Yet real‐world applications require *broad‐range, smooth, reversible*, and in situ modulation. On the other hand, mechanical metamaterials enable exotic static and dynamic mechanical properties beyond the limitations of natural materials [20, 26, 27]. Metamaterials with gear‐like and cam‐like metacells can offer versatile shape‐morphing modes [28, 29]. Gear‐based mechanical metamaterials [30] have recently emerged as a promising active paradigm, leveraging dynamic elements and hybrid strong‐weak coupling to control shape and achieve *broad‐range, smooth, reversible*, and in situ control over stiffness [30]. This idea has recently been extended to conceiving metamaterials for modulating longitudinal stiffness and vibration [31, 32], bending properties [33], Bragg bandgaps [32, 33], locally resonant bandgaps [34], isostatic unstability [35], and morphable shape [36]. However, three fundamental functionalities remain unrealized.

**FIGURE 1:**
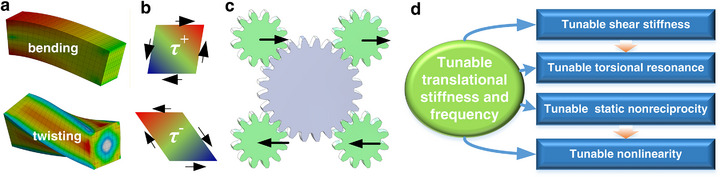
Conceptual design principles for tunable shear deformation. (a) Stress distribution in bending and twisting modes. (b) Sketch showing nonreciprocal shear states. (c) Design concept for metamaterials with tunable shear deformation. (d) Highlights of the design to enable extended tunability.

First, current designs focus almost exclusively on translational [30, 31, 32, 34, 35] or bending deformation [33], neglecting shear and torsional tunability—despite their dominance in bending and twisting responses of structures like beams, plates, and frames (Figure 1a).

Second, mechanical systems generally obey Maxwell–Betti reciprocity and symmetrical response. Mechanical metamaterials offer a platform for studying nonreciprocal mechanics [37, 38] and exploring new functionalities [38, 39]. In dynamics, combining linear and nonlinear metamaterials can trigger diode wave effects [40, 41] owing to the amplitude‐dependent bandgap shifts [39] and harmonics induced by nonlinearity [42]. Static nonreciprocity—asymmetric deformation under reversed loading (*F* ^±^ = *k* ^±^ *x* ^±^)—offers a route to unidirectional mechanics (Figure 1b) or non‐Newtonian behavior (unidirectional shock resistance) [43]. Although static nonreciprocity has been achieved via buckling [44, 45], clearance contact [46] or active control [47, 48, 49] and was realized in 3D metamaterial [50], there exists no feasible way up to now to *tuning* this property.

Third, beyond the resonant frequency and narrowband efficiency in linear dynamics, introducing nonlinearity into structures and metamaterials [51, 52] has been shown to produce robust and broadband effects for vibration attenuation [53, 54, 55], sound insulation [56], energy dissipation [57], bandgaps [58, 59], harmonics and solitons [60, 61]. However, in existing materials, nonlinear terms in stress‐strain relation (e.g., *σ* = *E*_1_ *ε*+ *E*_2_ *ε* ^2^+ *E*_3_ *ε* ^3^) are typically considered weak perturbations [62]—treated as negligible corrections to the dominant linear response (*σ* = *E*_1_ *ε*) under moderate strain. Critically, the nonlinear coefficients (*E*_2_, *E*_3_ …) are intrinsic material properties, dictated by the microstructural composition, thereby not independently controllable or requiring complex structures [32]. Consequently, despite the overwhelming desire to tune nonlinearity on demand and enable unprecedented balancing of linear and nonlinear physical processes [63, 64], this capability remains unrealized and poses a far greater challenge than tuning linear properties alone.

Motivated by these needs and challenges, here we propose gear‐based mechanical metamaterials that simultaneously achieve in situ, continuous, and broad‐range tunability of shear stiffness (accompanied with translational stiffness), along with static nonreciprocity (Figure 1d). These features allow for the design of adaptive structures such as resonators with broadly tunable frequencies in both translational and torsional modes. Crucially, we demonstrate that breaking static reciprocity provides a general mechanism to induce softening nonlinear dynamics, and that *tunable nonreciprocity* allows for precise control over nonlinear resonance profiles. Our work establishes a unified framework for multi‐functional metamaterial design, opening a new avenue to conceive adaptive materials and structures for next‐generation control systems.

## 2 Results

Although applicable to longitudinal deformation, we focus on shear‐dominated responses and torsional motion, which are essential to structural integrity yet historically underexplored in active metamaterials. Unlike axial tuning—governed by in‐line unit‐cell compliance—controlling shear requires the precise modulation of diagonal intercell coupling (Figure 1c). To address this, we introduce two gear‐based architectures, supported by analytical models that reveal the underlying mechanisms.

### 2.1 Taiji Gear‐Based Metamaterials with Smoothly Tunable Shear Modes

**FIGURE 2:**
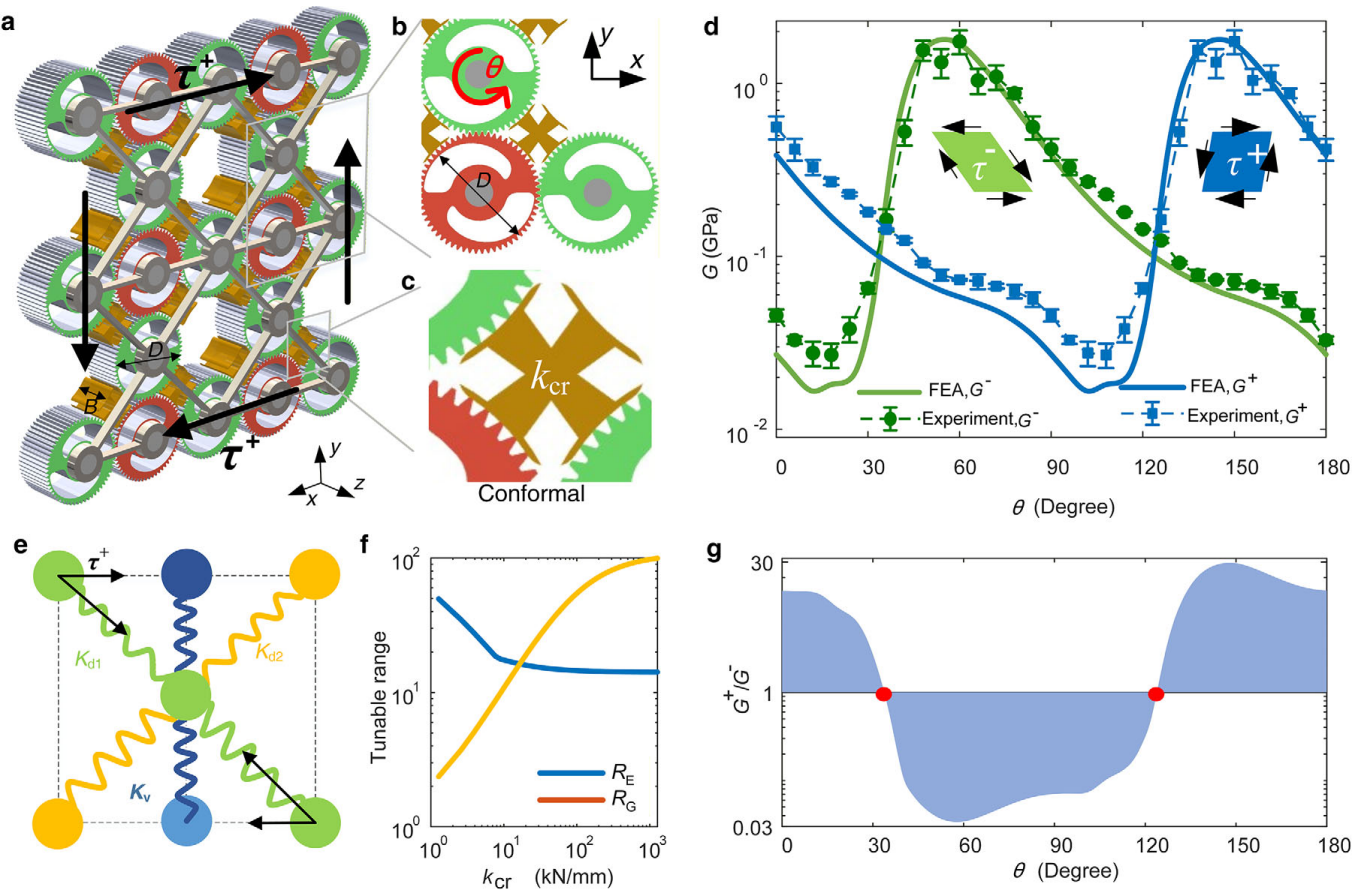
Tunable shear deformation in metamaterials based on Taiji gears. (a) Metamaterial configuration with inserted X‐shape elements giving a tunable shear modulus. The gears are identical, but its front and back planes are distinguished with green and red colors. (b) Meshing between neighboring gears. (c) The enlarged picture shows one of the inserted X‐shaped objects with flippers (the bronze part). (d) Measured and simulated shear moduli in positive *G* ^+^ and negative *G* ^−^ directions. Two parallelograms illustrate the nonreciprocal deformation under positive and negative shear stress *τ* ^+^ (*τ* ^−^). (e) Simplified mechanical model for the tunable and nonreciprocal properties. (f) Tunable ranges of Young's and shear modulus vary with the stiffness of the insert crosses *k*_cr_. (g) Ratio *G* ^+^/*G* ^−^ obtained from panel (d).

The first design employs planar gears with an intrinsic stiffness gradient (Figure 2a). Inspired by the Chinese *Taiji* (yin–yang) symbol, each “Taiji planar gear” incorporates S‐shaped elastic arms formed by removing two asymmetric segments from the gear body (Figure 2b; Figure S1). As demonstrated in tunable longitudinal stiffness [30], this gear layout offers a great stiffness gradient, enabling broad‐range variable stiffness from the thinnest to thickest positions.

The proposed metamaterial design is a gear array connected by two soft frames and the gears’ center shafts (Figure 2a). Neighboring gears are engaged together for smooth motion transmission under heavy load. The metamaterial arrays comprising tightly coupled Taiji gears can offer a large shear modulus for protecting the structure from failure owing to the shear‐interlock regime (Figure S1c), but their shear modulus is only narrowly tunable [30].

To unlock a wide tunable range, we introduce cavities that disrupt the shear interlock, permitting controlled interlayer sliding. The periodic metacell comprises a 3 × 3 gear cluster by removing the center gears from opposite sides. Into the cavities, we insert X‐shaped load‐transfer elements (bronze in Figure 2c), optimized with flappers that conformably contact three adjacent gears without inducing jamming. No more constrains are applied on them. These elements mediate diagonal force transmission across the array while stabilizing both shear and compressive deformations.

The metamaterial can be assembled with *n* × *n* metacells in Figure 2a, and the entire array can be actuated by rotating any single gear with angle *θ* (Figure 2b). Due to the Taiji gear's antisymmetric profile and the opposite rotation angles of the two engaged gears, each gear pair acquires a polarized mechanical phase with an offset angle. To maximize tunability, we configure all meshing pairs with zero angular offset and opposite rotation senses (Figure 2b), ensuring simultaneous alignment of their stiffest (or softest) states during actuation. In this case, the compression stiffness of a pair of elastic arms *k*_p_ equals the stiffness of a whole gear (Figure S1b).

To demonstrate the tunable equivalent shear modulus *G* arising from this design, we fabricate a sample consisting of copper gears with pitch diameter of *D* = 42 mm and a gear width of *B* = 20 mm (Methods, Figure S2). Finite element analysis (FEA) is used to calculate *G* by considering the contact between the meshing teeth. Within a small deformation range, the equivalent shear stress *τ* ^±^ induces shear strain *γ* ^±^, *τ* ^±^ *= G* ^±^ *γ* ^±^, where ± distinguishes the two opposite directions of shear stress (Figure 1b). Experiments on a 3 × 3 array confirm the model predictions.

To capture the essential physics, we develop an analytical model in which each gear pair is represented by a spring of stiffness *k*_p_ and consider the stiffness of the X‐shaped element *k*_cr_ (Figure 2e, Methods). Using steel crosses with *k*_cr_ = 1280 kN/mm (Figure 2d), this design shows smoothly tunable shear modulus over two orders of magnitude (0.02∼2.15 GPa, 107 times) from soft to rigid states within the whole tuning period, that is 0°<*θ<*180°.

Remarkably, the architecture also enables **tunable static nonreciprocity** in shear. Under positive or negative shear stress *τ* ^+^ (*τ* ^−^), load paths follow distinct diagonal chains (*K*_d1_ (*K*_d2_) in Figure 2e). Analytical expressions (Methods) yield:

(1) $$G^{+}\left(\theta \right)=\frac{k_{p}\left(\theta -45^{\circ }\right)k_{\mathrm{cr}}}{4B\left[k_{p}\left(\theta -45^{\circ }\right)+k_{\mathrm{cr}}\right]},G^{-}\left(\theta \right)=\frac{k_{p}\left(\theta +45^{\circ }\right)k_{\mathrm{cr}}}{4B\left[k_{p}\left(\theta +45^{\circ }\right)+k_{\mathrm{cr}}\right]}$$

The above expressions imply *G* ^+^(*θ*) = *G* ^−^(*θ‐*90°). Consequently, reciprocity *G* ^+^ = *G* ^−^ occurs only at two isolated points in a tuning period, while nonreciprocity occurs across the tuning cycle (Figure 2g). The nonreciprocity strength *G* ^+^/*G* ^−^ is smoothly tunable from 0.03 to 30. *G* ^+^/*G* ^−^<1 and *G* ^+^/*G* ^−^>1 correspond to the two polarities of nonreciprocity, which affect the static deformation, generating very soft yet strong states in opposite directions.

Notably, the compressive Young's modulus in the vertical axis, *E*_y_, also becomes synchronously tunable. The analytical theory predicts

(2) $$E_{y}\approx G_{g}^{+}+G_{g}^{-}+k_{p}/2B$$

The tunable ranges of the Young's modulus, *R*_E_ *= E*_max_/*E*_min_, and that of the shear modulus, *R*_G_ = *G*_max_/*G*_min_, vary oppositely with the stiffness of the X‐shaped cross *k*_cr_ (Figure 2f). For the experimental sample, *R*_E_ can be tuned up to a broad range of 40 times, demonstrating exceptional multi‐axial adaptability.

Together, the broad, continuous tunability of the shear and longitudinal stiffnesses, combined with large, polarized, and smoothly adjustable nonreciprocity, establishes a new class of mechanical metamaterials with exotic mechanical properties.

### 2.2 Planetary Gear‐Based Metamaterials with Smoothly Tunable Shear Modes

**FIGURE 3:**
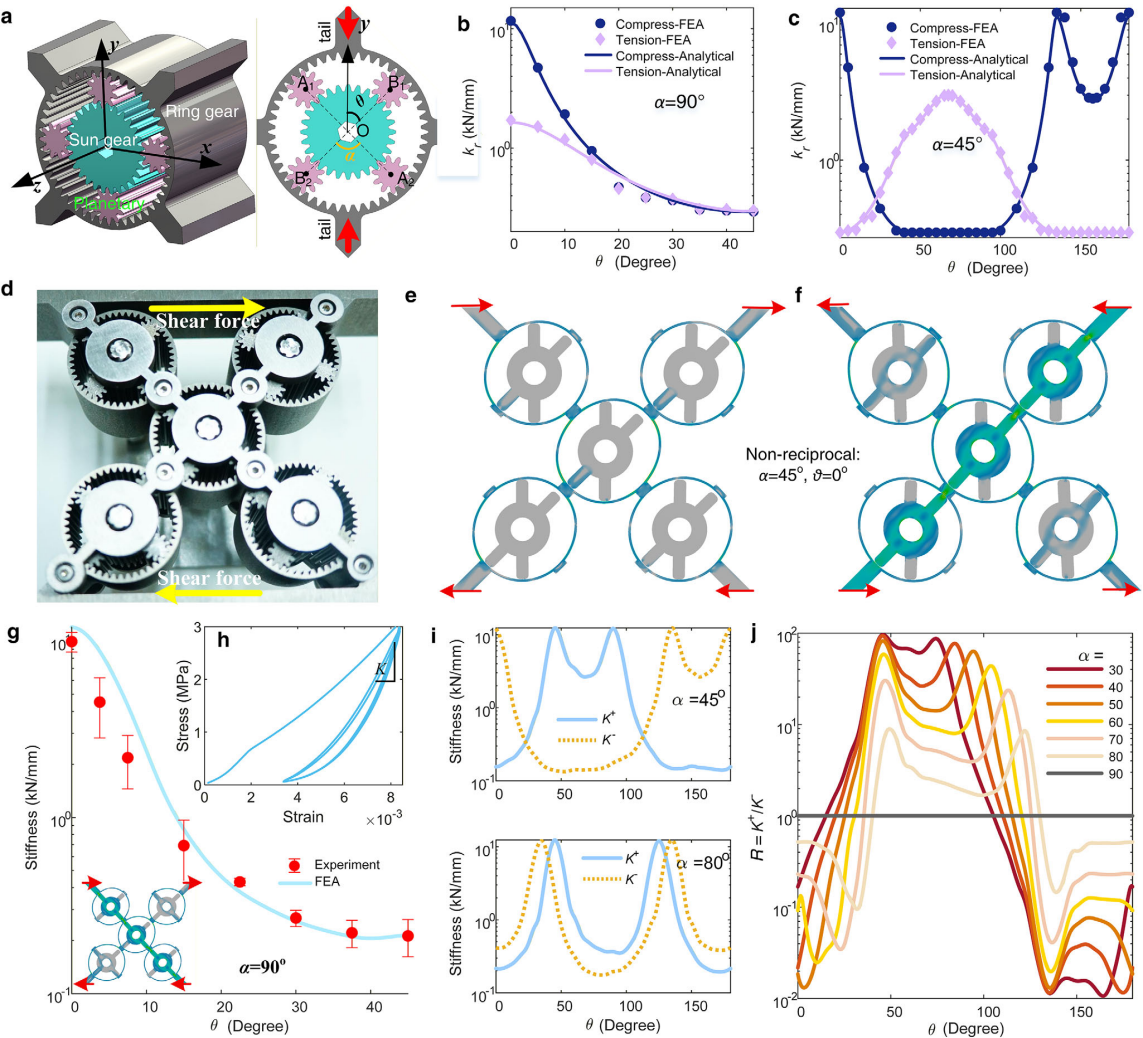
Tunable shear deformation in metamaterials based on planetary gears. (a) Planetary gear assembly using as a unit of following designs. (b,c) Compressive and tensile stiffness of a steel unit when applying loads on the tails for *α* = 90° and *α* = 45°. (d) Picture of the whole metamaterial sample consisting of five units. (e, f) Typical nonreciprocal deformation modes for *α* = 45°, *θ* = 0°. (g) Experimental and FEA shear stiffness of the whole metamaterial sample for *α* = 90°. The inserted panel (h) shows the cyclic loading process in experiment. (i) Positive and negative stiffness for typical cases with *α* = 45° and *α* = 80°. (j) Shear stiffness ratio *R* = *k* ^+^/*k* ^−^ between positive and negative loading cases, indicating the strength of nonreciprocity.

While the Taiji gear architecture delivers exceptional tunability, its reliance on boundary shaft loading limits its applicability in scenarios where distributed or surface‐applied forces are required. To overcome this constraint, we introduce a planetary gear–based metamaterial, in which each unit cell is built from compact, self‐contained planetary gear assemblies that enable intrinsic, load‐path–agnostic shear tunability (Figure 3d).

**FIGURE 4:**
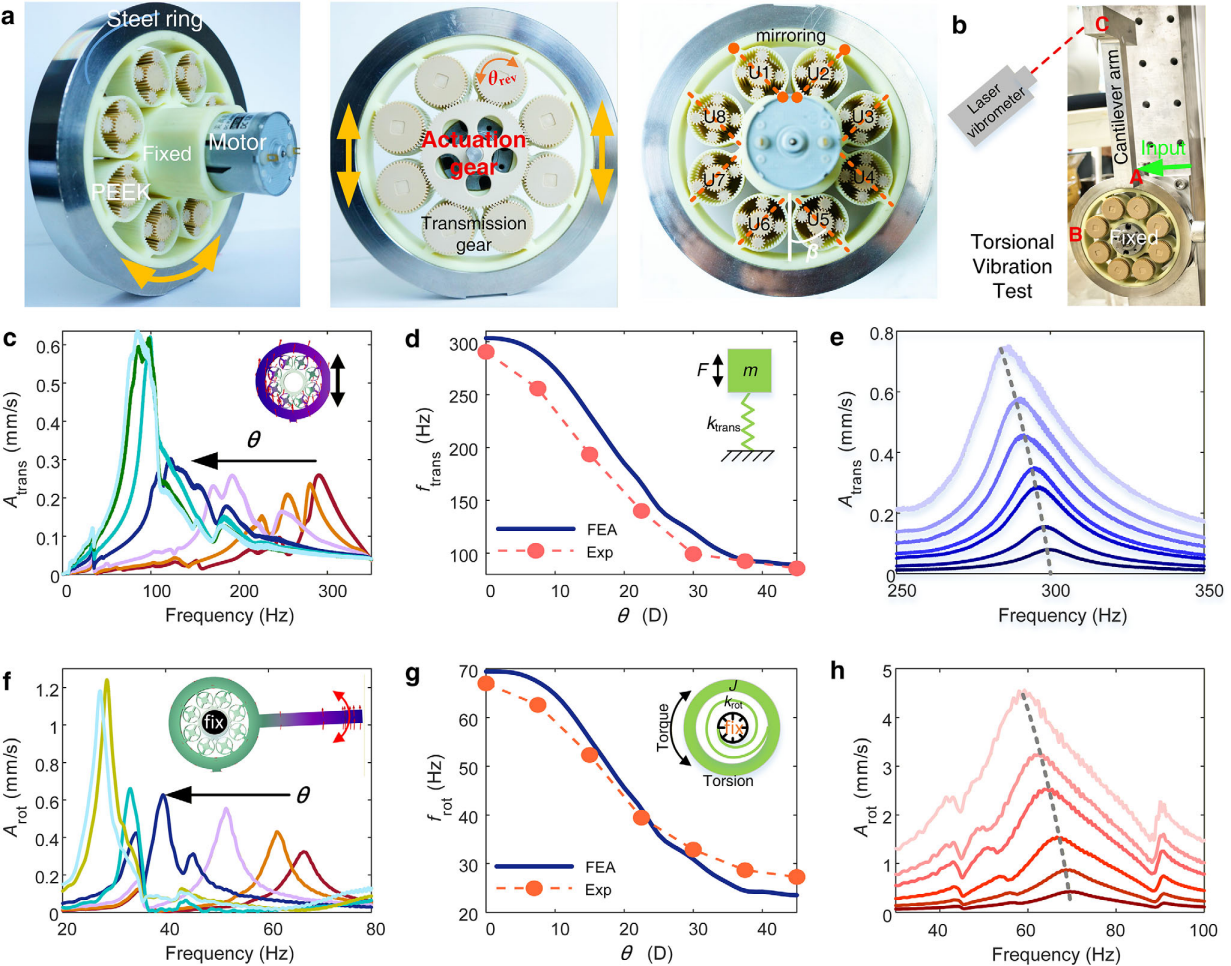
Variable‐frequency resonators based on gear metamaterials. (a) Pictures and structures of metamaterial resonator—a gear‐integrated ring. (b) Methods for testing torsional vibration by including a cantilever arm to amplify the torsional vibration. This arm is excluded when measuring the pure transverse vibration. More experimental details are explained in Figure S4. (c) Transverse vibration response spectra under different rotation angle *θ*. (d) Variation of transverse resonant frequency *f*_trans_ against *θ*. (e) Variation of transverse response when increasing the incident amplitude, showing weak geometrical nonlinearity. (f,g,h) Similar to contents in (c,d,e), showing the torsional vibration response and frequency *f*_rot_ vary with *θ* and amplitude.

The metacell is a X‐shaped structure, in which the center point and the four arms are linked by five identical planetary gear assemblies. Every assembly comprises a sun gear, a ring gear, and four planetary gears (Figure 3a). *r*_sun_ and *r*_pl_ are the pitch radii of the sun and the planetary gears, respectively; the pitch radius of the ring gear is *r*_ring_ = *r*_sun_+2*r*_pl_. The four planetary gears (A_1_A_2_ and B_1_B_2_) are center‐symmetrical. The installation angle between the two pairs, ∠A_1_OB_1_ = *α*, governs the global properties. One can actuate the whole architecture by rotating every sun gear individually, or connecting every sun gear to a transmission gear and then engaging all transmission gears together, so that one can actuate the whole architecture by rotating only one gear, as what have been done in Figure 4.

When applying tensile or compressive loads on the cell's tails, the ring undergoes bending deformation and squeezes the inner gears. If all inner gears are absent, the stiffness of the pure ring (of thickness *t* and width *b*) is *k*_0_ = 4π*E*_s_ *I*/*r*_ing_^3^(π^2^‐8), where *E*_s_ denotes ring's Yong's modulus, and *I* = *bt* ^3^/12 (Methods). When inner gears are present, the ring acts as a bending arm, and the planetary gears act as its fulcrums. When changing the revolution angle of the planetary gears *θ* = ∠B_1_Oy by rotating the center sun gear, the bending stiffness of the ring *k*_r_ is changed because its fulcrums are shifted. The bending stiffness *k*_r_ depends on the contact state between the planetary gears and the ring under compression or tension, because the deformed ring may either squeeze the inner gears or detach with them (Figure 3e). The height of the gear tooth avoids the ring to completely detach from the planetary gears if they lose contact.

In most case within 0≤*θ*≤180°, only one pair of the planetary gears (A_1_A_2_ or B_1_B_2_) is active under load; the other pair loses contact with the ring. The two pairs switch at critical angles. Moreover, both pairs can be inactive in some cases. For example, if A_1_A_2_⊥B_1_B_2_ (i.e., *α* = 90°), when A_1_A_2_ is active under compression, B_1_B_2_ will be active under tension and vice versa, but both A_1_A_2_ and B_1_B_2_ are inactive at the rotation angle *θ =*45°, where the minimal stiffness min(*k*_r_) = *k*_0_ is achieved (Figure 3b).

We develop an analytical model in (Section S4 and Figures S7–S11) that captures this behavior through angle‐dependent functions: $k_{r}^{\mathrm{comp}}\left(\alpha ,\theta \right)$ for compression and $k_{r}^{\mathrm{tens}}\left(\alpha ,\theta \right)$ for tension. For *α* = 90°, we have

(3) $$k_{r}^{\mathrm{comp}}=\frac{k_{0}+k_{f}}{k_{0}+k_{f}g_{1}\left(\theta \right)}k_{0},\;k_{r}^{\mathrm{tens}}=\frac{k_{0}+k_{f}}{k_{0}+k_{f}g_{2}\left(\theta \right)}k_{0}$$

here, *k*_f_ denotes the effective fulcrum stiffness in the case of squeezing of a planet–sun–planet assembly; *g*_1_(θ) and *g*_2_(θ) are functions of the rotation angle *θ*, and 0≤g_1_≤1, 0.16≤g_2_≤1 (Methods). If *k*_0_<<*k*_f_, max($k_{r}^{\mathrm{tens}}$)<<max($k_{r}^{\mathrm{comp}}$) and the maximal tunable range of tensile stiffness is 6.25 times.

We fabricate a steel sample for experimental demonstration and verification. As confirmed by the FEA at *α* = 90° and *α* = 45°, the analytical results agree with FEA (Figure 3b,c; Figure S12). The compressive tunability, *R*_com_ = 1+*k*_f_/*k*_0_, is independent of the installation angle *α*, while tensile tunability *R*_tens_ peaks at *α* = 50° (Figure S13). Crucially, tension–compression asymmetry emerges across most of the tuning cycle (Figure 3b,c)—a form of uniaxial nonreciprocity that is itself continuously adjustable.

We assemble the full X‐shaped metacell with all five units sharing identical phase angle *α* and actuation angle *θ* (though independent control is feasible). FEA incorporating realistic gear contact effects yields convergent predictions for the equivalent shear stiffness *K* (Figure 3d). Cyclic shear experiments are conducted to measure *K* within small deformation (Figure 3h). The first uploading process leads to tiny and unrecoverable deformation, attributing to the gear meshing contact. Subsequent cycles are fully recoverable and repeatable, giving the experimentally measured *K* at relatively large shear force, that is, the data from the first uploading process are not used to calculate *K*. The meshing contact between the gear teeth leads to frictional dissipation within small deformation. Experimental results at different rotation angles evidence a repeatable, large yet smooth tunable range of 50 times, in excellent agreement with simulation (Figure 3g).

Interestingly, we also observe direction‐dependent stiffness (*K* ^+^, *K* ^−^) under positive or negative shear loads for this design. For *α* = 90°, symmetrical contact states preserve reciprocity and always give *K* ^+^(*θ*) = *K* ^−^(*θ*) (Figure S3). But for *α* ≠ 90°, the contact states in the planetary units along the two diagonal directions are different under positive and negative shear, breaking reciprocity (i.e., *K* ^+^≠*K* ^−^). For example, for *α* = 45° and *θ* = 0, the positive loading corresponds to a soft state (Figure 3e) while the negative loading encounters stiff diagonal trace (Figure 3f), yielding a nonreciprocity ratio as high as max(*K* ^+^/*K* ^−^) = 80. This nonreciprocity doubles the tuning period of stiffness to 180°, evidenced by the two peaks in a period (Figure 3i). To avoid the contact between the two planetary gears inside a unit, we require *α*>30°. Within the range of 30°<*α*<90°, a smaller angle *α* gives stronger larger nonreciprocity (Figure 3j). At *α* = 30°, *R = K* ^+^/*K* ^−^ vary smoothly from 0.01 to 100, signaling a large tunable range of nonreciprocity and polarity.

This planetary architecture thus achieves three simultaneously tunable functionalities: (1) broad‐range shear stiffness, (2) tension–compression asymmetry, and (3) large, smooth, and polarity‐switchable static nonreciprocity.

### 2.3 Resonators with Variable‐Frequency Enabled by New Metamaterials

The tunable, nonreciprocal metamaterials discussed above hold promise in controlling the deformation of solids, structures, machines, and robots. As an illustrative example, we design a multifunctional resonator whose transversal and torsional resonant frequencies can be continuously tuned—a capability hardly achievable in conventional adaptive systems.

The resonator features a gear‐integrated ring (Figure 4a), where a steel ring serves as the “mass” *m* or “inertia” *J*. Its “variable‐stiffness spring” consists of *n* planetary assemblies periodically inserted between inner and outer rings (Figure 4b). The gear ring is printed using resin, while the planet and sun gears are fabricated using PEEK polymer, which are assembled with the ring. Moreover, a transmission gear is mounted on every sun gear (Figure 4a) that meshes with a center actuation driven by a motor, enabling synchronous control of all units via a single input. We adopt a small interference fit between inner gears and the ring gear, which can eliminate the clearance nonlinearity within small deformation and maintain high reliability under large deformation (i.e., avoid disengagement between them).

The orientation of the connection tails—which anchor each planetary unit to the inner and outer rings—dictates the coupling between deformation modes. In our prototype, eight units (U1–U8) are grouped into four pairs (Figure 4a), with the two tails in each pair arranged perpendicularly to form a “V” geometry. For simplicity without losing generality, all units have identical installation angle *α* = 90° and the same phase. We fabricate samples with *r*_sun_ = 12 mm, *r*_pl_ = 6 mm and *t* = 0.55 mm. The mass of the steel ring is *m* = 1 kg, whose rotation inertia *J* depends on its shape. *J* = 0.01 kg·m^2^ here. We calculate and measure its eigen modes by fixing the inner ring (Figure 4b). The first‐order transverse and torsional resonant frequencies (*f*_trans_ and *f*_rot_) are

(4) $$f_{\mathrm{trans}}=\sqrt{\frac{k_{\mathrm{trans}}}{m}}/2\pi ,f_{\mathrm{rot}}=\sqrt{\frac{k_{\mathrm{rot}}}{J}}/2\pi$$

where *k*_trans_ and *k*_rot_ are the total transverse and torsional stiffness coefficients, respectively. Experiments confirm smooth and in situ tunability across broad ranges: *f*_trans_ can be smoothly tuned by 3.3 times from 90 Hz to 300 Hz (Figure 4c,d); *f*_rot_ by 2.8 times from 25 to 70 Hz (Figure 4f,g).

Existing methods for tunable frequency generally offer narrow tunable range, rely on large deformation and or operate only in translational modes. Our method offers broader tunable range, smooth varying process, and avoidance of global deformation (in situ tunability), enabling true real‐time adaptability.

Though both transverse and torsional vibration modes are tunable, their mechanisms are different. During transverse vibration, units on opposite sides experience symmetric compression and tension (e.g., U1–U4 compressed, U5–U8 tensed). Under torsional vibration, alternating units compress and stretch simultaneously (e.g., U1, U3, U5 and U7 are compressed and the rests are stretched). In both cases, the bidirectional stiffness coefficients are identical. This ensures linear dynamics, whose frequency is amplitude‐independent. In practice, when increasing the incident amplitude, experiments demonstrate a slight shift of *f*_trans_ (*f*_rot_) toward low frequency by about 5% (10%) (Figure 4e,h). Such weak nonlinear effect only originates from the large geometrical deformation. Greater shift is observed for *f*_rot_ because the transverse motion is amplified by the large inertia *J* (Figure S5).

### 2.4 Programable Nonlinear Resonator Enabled Nonreciprocity

Nonlinear resonators are pivotal in broadband vibration reduction, energy absorption, sound insulation, and outperform linear counterparts by adapting their response to excitation level. Among them, softening‐nonlinear resonators, whose resonant frequency decreases with excitation amplitude, are especially valuable for low‐frequency control. However, their utility has been limited by fixed and hardly controllable nonlinearity strength. We demonstrate a general principle: static nonreciprocity in Figure 3 enables the remarkable tunability of strength of softening nonlinear resonators.

**FIGURE 5:**
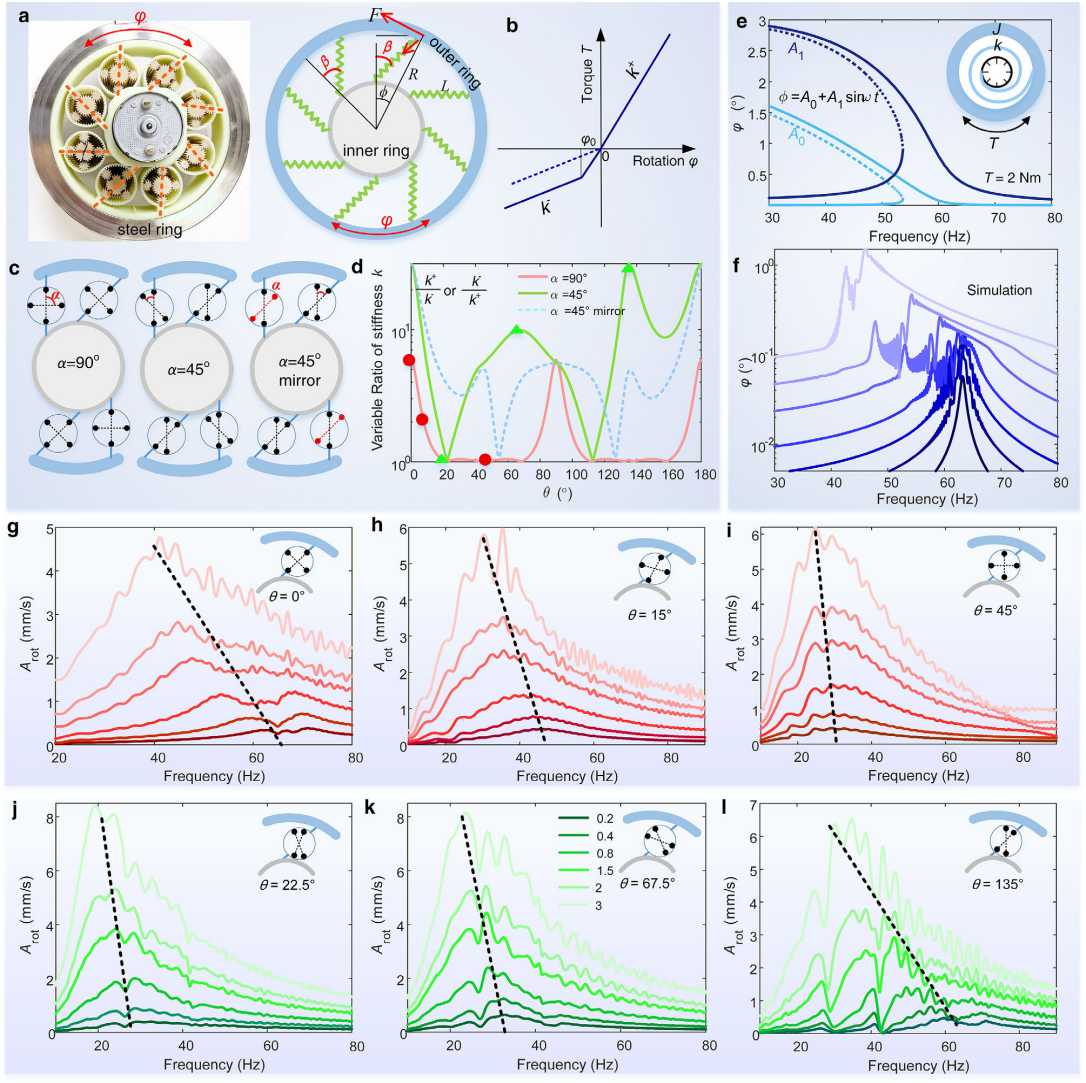
Tunable nonlinear dynamics enabled by nonreciprocity. (a) Components and equivalent model of nonreciprocal gear ring. (b) Piecewise linear relationship between the torque and dynamic rotation angle *φ*. (c) Three cases with different initial angles *α* to analyze properties. (d) Ratios of rotation stiffness of the three cases in (c) vary with rotation angle *θ*. Ratio *R* = max(*k* ^+^/*k* ^−^, *k* ^−^/*k* ^+^). (e) Amplitude‐frequency bifurcation curves of the nonlinear torsional resonator, with solution assumed as *φ* = *A*_0_+*A*_1_sin*ωt*. (f) Typical numerical results calculated by the integration under increasing torque amplitudes *T*. In (e,f), *α* = 90°, *θ* = 0°. (g‐l) Experimental torsional vibration responses varying with increasing amplitudes for typical cases with different stiffness ratios labeled with points in (d). (g,h,i) Results in the pattern *α* = 90°: *θ* = 0°, *θ* = 15°, *θ* = 45°. Corresponding transversal (not torsional) responses of the steel ring are presented in Figure S5. (j,k,l) Results in the pattern *α* = 45°: *θ* = 22.5°, *θ* = 67.5°, *θ* = 135°. The rotation phase of one unit is presented in every panel in (g‐l). The incident amplitude is changed by 10 times in (g‐i), and is changed by 15 times in (j‐l).

We still take the gear ring as a platform, now reconfigured to break rotational symmetry. Shear nonreciprocity behaves as the clockwise and anticlockwise stiffness (*k* ^+^ and *k* ^−^) for the rotation. One can hardly break this symmetricity in the gear ring consisting of V‐shaped unit pairs in Figure 4. To break the rotational symmetricity, we align all connection tails at a uniform oblique angle relative to the radial direction (Figure 5a). We set the eccentric angle *β* = 45°. Thus, all oblique units are synchronously compressed or tensed in rotation, inducing asymmetrical torsional stiffness.

Based on the single‐unit model from Figure 3 and Figure S14, we further establish the analytical model for the torsional stiffness of the whole gear ring, as detailed in Section S5. Similar with Figure 3, torsional stiffnesses *k* ^+^ and *k* ^−^ depend on the arrangement angle *α* and can be tuned by the inner actuation angle *θ* (Figure S15). We compare three configurations: *α* = 90°, *α* = 45°, and “α = 45° mirror”, as shown in Figure 5c. Every unit has an identical phase angle for the former two cases. In the “α = 45° mirror” case, the two pairs of the planetary gears in neighboring units switch their position though their angles are identical (*α* = 45°).

We quantify nonreciprocity via the ratio *R* = max(*k* ^+^/*k* ^−^, *k* ^−^/*k* ^+^) based on an analytical model. The nonlinear strength of the resonator can also be manifested by *R*. As shown in Figure 5d, *R*(*θ*) is highly sensitive to microstructural arrangement. Specifically, for *α* = 90°, *R*(*θ*)>2 only in 30% of the tuning range *θ*∈[0 π]. In contrast, for *α* = 45°, this range reaches 95%, and the peak value becomes much larger, evidencing strong, sustained nonlinearity across nearly the entire actuation cycle.

The torsional vibration of the inertia *J* depends on a piecewise‐linear function (Figure 5b)

(5) $$J\ddot{\varphi }+c\dot{\varphi }+F\left(\varphi \right)=T,\;F\left(\varphi \right)=\left\{\begin{array}{c} k^{+}\varphi ,\;\;\;\;\;\;\;\;\mathrm{for}\;\varphi \geq \varphi_{0}\\ k^{-}\varphi +\left(k^{+}-k^{-}\right)\varphi_{0},\mathrm{for}\;\varphi <\varphi_{0} \end{array}\right.$$

Here, *T* denotes the input torque; *φ*_0_ denotes the pre‐deformation angle induced by the interference‐fit assembling process; *c* is the damping coefficient. As evaluated from experiments, *φ*_0_ = −0.6° and *c* = 0.05 N·m·s/rad here.

By fitting this piecewise function with a polynomial equation, we can obtain the steady response using the harmonic balance method (Methods, Figure S17), where the solution is assumed as *θ* = *A*_0_+*A*_1_sin*ωt*. Due to the unsymmetrical piecewise stiffness, the solution has a nonzero offset *A*_0_. The resulting amplitude–frequency curves exhibit classic softening behavior, bending toward lower frequencies and displaying bifurcation and jump phenomena (Figure 5e). Direct time‐domain simulations under swept‐sine excitation confirm these trends (Figure 5f). Jumping behaviors appear due to the switching between two bifurcation branches. Strong nonlinearity may induce 50% shifting of the peak frequency. Vibration experiments for *α* = 90° and *α* = 45° are conducted to demonstrate the tunable nonlinear responses beyond the tunable resonant frequency. We observe the frequency shifting by increasing the excitation amplitude for a specified angle *θ* by more than 10 times. The experimental results in Figure 5g–l align to the numerical results under the same input (Figure S16).

For *α* = 90°, the nonreciprocal ratio *R* decreases from 6 to 1 with *θ* increasing from 0 to 20° (Figure 5d), indicating weakened nonlinearity for larger *θ*. This is demonstrated by the 40% frequency shifting at *θ* = 0° and 30% shifting at 15° (Figure 5g,h); *R*≈1 in *θ*∈[20°, 45°], implying a quasi‐linear state, which is also demonstrated by the negligible frequency shifting at *θ* = 45°in Figure 5i.

For α = 45°, we pick three cases *θ =*22.5°, 67.5°, and 135°, where *R* = 1, 10 and 30, respectively (Figure 5d). The experimental spectra at *θ =*22.5° (Figure 5j) show negligible frequency shift when increasing the amplitude, confirming the quasi‐linear state; peak frequencies at *θ =*67.5° (Figure 5k) and 135° (Figure 5l) are shifted by 30% and 55%, respectively, demonstrating strong and enhanced nonlinear effects.

The tunable resonant frequencies at low input amplitude (linear response) are consistent with the theoretically predicted trends in terms of stiffness max(*k* ^+^, *k* ^−^), and the frequency shifting induced by amplitude (nonlinear response) is consistent with the trends of nonreciprocal ratio *R*. This dual correspondence confirms that static nonreciprocity serves as a continuous knob for nonlinear dynamic responses.

## 3 Conclusions and Discussions

Adaptive mechanical systems—from resonators to intelligent structures—demand materials and structures that can reconfigure stiffness, dynamics, and symmetry on demand. Conventional tunable architectures are largely confined to translational deformation, narrow range, discontinuous response or reliance on large‐strain actuation and linear dynamics. This paper proposes a novel gear‐based design paradigm of mechanical metamaterials that offer exceptional tunability for torsion, nonlinearity, and nonreciprocity—a capability demonstrated through two complementary designs: Taiji planar gears and planetary gear assemblies. Our methods offer in situ and continuously tunable shear stiffness over 100 times, exemplified by the design of mechanical resonators with broadly variable frequencies for both translational and torsional motion modes.

Beyond stiffness control, our designs break the static shear reciprocity when subject to positive and negative loads, owing to the asymmetrical bending arm in Taiji gear or the asymmetrical contact states in planetary gear assemblies. The static nonreciprocity is also smoothly tunable by over 100 times. Crucially, we show that such static asymmetry directly governs dynamic nonlinearity: by modulating the degree of nonreciprocity, we can continuously program structural (exemplified by a resonator) response from linear to strongly softening nonlinear (with great resonance shifts). Although existing studies have demonstrated that static nonlinearity can induced dynamic nonreciprocity [42], here we demonstrate the inverse: static nonreciprocity can be a precise control knob for triggering dynamic nonlinearity, a high‐order deformation that was hardly controllable. This establishes a bi‐pathway influences between nonlinearity and nonreciprocity. Reliable analytical models are established to underpin the design and elucidate the underlying mechanisms.

In conclusion, this work bridges a critical gap in the design of mechanical metamaterials, adaptive structures, and resonators by enabling simultaneous, independent control over translational and torsional stiffness (and thus resonance frequency), static nonreciprocity, and the transition between linear and nonlinear dynamics. The underlying principles are scalable, material‐agnostic, and compatible with compact, low‐power actuation, making them highly suitable for real‐world engineering applications—from adaptive deformation systems and broadband vibration isolation to the manipulation of linear and nonlinear elastic waves.

Beyond the demonstrated example using tunable resonator—a universal mechanical circuit element—this platform unlocks a rich landscape of advanced functionalities. Static nonreciprocity paves the way to design elastic or acoustic wave diodes for directional wave transport [38], and to achieve topological mechanics [49, 65] for controlling deformation/damage routes and stiffness under different loading directions; tunable torsion enables morphing structures and soft robotic actuators with on‐demand twisting capabilities [2]; periodic linear–nonlinear hybrid resonators can be engineered into multifunctional acoustic and seismic metastructures [51, 66, 67]; and the precisely controllable nonlinearity offers a pathway toward mechanical computing [1, 68] and analog signal processing [69]. Though including contact interfaces inside metamaterials can present nonreciprocity [46], one can conceive principles to make the tunable deformation symmetrical [32], thereby achieving linear dynamics. Together, these capabilities position gear‐based active metamaterials as a foundational toolkit for next‐generation adaptive and intelligent mechanical systems. Finally, while remaining challenging, assembling gears into 3D mechanical metamaterials is possible, in a way similar to the design of layered gear‐based meta‐beam and plate frameworks (quasi‐3D structures) with tunable bending properties [33].

## 4 Methods

### 4.1 Model Parameters

Parameters for metamaterials based on Taiji gear are shown in Figure S1. In experiments, the gear has 60 teeth, and its pitch diameter is *D* = 42 mm. They are made of copper. The solid part of the circular gear has two shoulders and two elastic arms, which is realized by removing two irregular‐shape holes inside the gear body. In a meshing pair, their spin directions are opposite, but the patterns on the front and back planes of Taiji gear are also opposite. Thus, a meshing pair generates polarity. Here we use the positive polarity in which a pair of meshing Taiji gears has opposite spiral directions, making sure that the thinnest (and thickest) positions of the two gears meet in rotating, offering the maximal tunable range.

In planetary gear systems, the planetary gears undergo self‐rotation and revolution round the sun gear. The revolution and self‐rotation angles are

$$\theta_{\mathrm{rev}}=\frac{\theta_{\mathrm{sun}}r_{\mathrm{sun}}}{2\left(r_{\mathrm{sun}}+r_{\mathrm{pl}}\right)},\;\theta_{\mathrm{self}}=-\frac{\theta_{\mathrm{sun}}r_{\mathrm{sun}}}{2r_{\mathrm{pl}}}$$

where *r*_sun_ and *r*_pl_ are pitch radii of the sun and planetary gears, respectively; the pitch radius of the ring gear is *r*_ring_ = *r*_sun_+2*r*_pl_. In the whole paper, the actuation angle for characterizing the properties denotes *θ* = *θ*_sun_.

In this paper, all planetary gears have *r*_sun_ = 12 mm, *r*_pl_ = 6 mm, *r*_ring_ = *r*_sun_+2*r*_pl,_ and width *b* = 20 mm. Moreover, their gear teeth are of 0.5 module, thus, the ring has 48 teeth. The samples in Figure 3 are made of steel of Young's modulus *E* = 200 GPa and Poisson's ratio 0.3, and their ring's thickness is *t* = 0.5 mm. The ring gears in Figures 4 and 5 are printed with resin of Young's modulus *E*≈2 GPa and thickness *t* = 0.65 mm, while their planet and sun gears are fabricated using PEEK polymer of *E*≈4 GPa because stiffer polymer for inner gears can maintain a broader tunable range.

### 4.2 Experiment Tests

For the sample consisting of Taiji gears in Figure 2, we test a metacell consisting of 7 Taiji gears, steel crosses and steel frames to obtain the tunable and nonreciprocal shear modulus. The installing manner of the sample are shown in Figure S2. Compressive load from the testing machine transfers to the sample through a pair of connecting shafts whose overall stiffness is *K*_shaft_. The two shafts have considerable influence on the testing result. The inset on the bottom right corner shows the mechanical model. The real stiffness of the metamaterial is *K*_meta_. 1/*K*_test_ = 1/*K*_shaft_+1/*K*_meta_. We adopt this mechanical model to correct the measured result to obtain the real shear modulus of the metamaterial. For the steel sample made of planetary gears, we directly apply shear force on the two sides of the sample, as shown in Figure 3.

Vibration test methods are shown in Figure S4. we fix the inner ring of the whole gear ring and excite the external steel ring to obtain the vibration. Laser vibrometers are adopted to measure the responses at excitation and response points. When measuring the torsional vibration, a cantilever arm is fixed on the external steel ring to amplify the rotation angle and distinguish the transverse and torsional resonant frequencies. Our experimental apparatus has *J* = 0.01 kg·m^2^.

### 4.3 Equivalent Method for the Metamaterial Based on Taiji Gears

The metacells shown in Figure 2 in the main text can be equivalent to the discrete model shown in Figure 2e. The inserted crosses influence not only the modulation of shear modulus *G* but also the Young's modulus *E*_y_. For the three central gears along *y* direction, *K*_v_(*θ*) = *k*_p_/2. The entire stiffnesses in two diagonal directions are:

$$K_{d1}\left(\theta \right)=k_{p}\left(\theta -45^{\circ }\right)k_{\mathrm{cr}}/2\left[k_{p}\left(\theta -45^{\circ }\right)+k_{\mathrm{cr}}\right]$$

$$K_{d2}\left(\theta \right)=k_{p}\left(\theta +45^{\circ }\right)k_{\mathrm{cr}}/2\left[k_{p}\left(\theta +45^{\circ }\right)+k_{\mathrm{cr}}\right]$$

Here *θ* denotes the gear rotation angle respective to its coordinate origin; *k*_cr_ denotes the stiffness of the cross object in the diagonal direction. In the shear stress state (Figure S6), only one arm of the cross is loaded because the other arm is not in contact with any gear. The geometrical deformation relationship is

$${\left(\sqrt{2}a_{y}-\Delta_{g}\right)}^{2}={\left(a_{x}-u\right)}^{2}+a_{y}^{2}$$

where *a_x_* = 2*D* and *a*_y_ = 2*D* denote the lattice constant; *u* = *γa*_y_ is the relative displacement between the two rows of gear centers; *γ* and Δ_g_ denote the shear strain and the deformation of the diagonal gears. By neglecting the nonlinear term, one obtains

$$\Delta_{g}=\frac{\sqrt{2}u}{2}$$

Therefore, the shear force supported by diagonal gears is

$$F_{x1}=\frac{\sqrt{2}K_{d1}\Delta_{g}}{2}=\frac{K_{d1}u}{2}$$

$$F_{x2}=\sqrt{2}K_{d2}\Delta_{g}/2=\frac{K_{d2}u}{2}$$

When applying shear stress in the positive direction *τ* ^+^, the shear force is supported by the diagonal gears connected by crosses 1. Therefore,

$$G_{g}^{+}\left(\theta \right)=F_{x1}/a_{x}B\gamma =K_{d1}\left(\theta \right)/2B$$

On the contrary,

$$G_{g}^{-}\left(\theta \right)=F_{x2}/a_{x}B\gamma =K_{d2}\left(\theta \right)/2B$$

Therefore, $G_{g}^{+}$(*θ*)= $G_{g}^{-}$(*θ‐*90°).

However, when applying the compressive stress *σ*_y_, all gears take part in supporting the stress. In this case, the deformation of the center gear becomes complicated. By using the boundary conditions *ε*_x_ = 0, *γ* = 0 and *v* = *ε*_y_ *a*_y_, we obtain the equivalent Young's modulus in this case:

$$E_{y}\;\approx \;K_{v}/B+\left[K_{d1}\left(\theta \right)+K_{d2}\left(\theta \right)\right]/2B+E_{f}=G_{g}^{+}+G_{g}^{-}+k_{p}/2B$$

Therefore, the compressive Young's modulus *E*_y_ is synchronously tunable.

### 4.4 Stiffness of an Empty Circular Ring

The ring gear in the planetary assembly can be treated as an empty circular ring. Here, *r* is the centroid radius of the ring, *E* is the Young's modulus of the ring material, and *I* is the moment of inertia of the ring cross‐section, calculated as *I* = 12*bt* [3], where *b* is the width of the ring cross‐section and *t* is the thickness of the ring cross‐section. When applying a radial force *F* at the two tails on the ring (see Figure 3a), the radial displacement Δ is indued. The linear stiffness of the empty ring writes

$$k_{0}=\frac{F}{\Delta }=\frac{4EI\pi }{r^{3}\left(\pi^{2}-8\right)}$$

The whole derivation procedure is detailed in Section S4.1. It is obtained by establishing the equilibrium equations of force and torque.

### 4.5 Stiffness of a Single Planetary Gear Unit

The stiffness of the planetary assembly, *k*_r_, depends on the initial installing angle *α*. As the force transmission manner changes with the rotation angle *θ* and switches at critical rotation angles, one can hardly obtain a general expression for *k*_r_(*α, θ*). Here we distinguish the expressions under different angles *α*. The whole derivation procedure is detailed in Sections S4.2 and S4.3. Let's take α = 90° as example to show the results. In the case of α = 90°, the equivalent compressive stiffness of the ring is:

$$k_{r}^{\mathrm{comp}}=\frac{\left(k_{0}+k_{f}\right)k_{0}}{k_{0}+k_{f}g_{1}\left(\theta \right)}$$

The equivalent tensile stiffness of the ring is:

$$k_{r}^{\mathrm{tens}}=\frac{\left(k_{0}+k_{f}\right)k_{0}}{k_{0}+k_{f}g_{2}\left(\theta \right)}$$

Here, *k*_f_ denotes the total compression stiffness of a fulcrum, which is formed by a sun gear and a pair of planetary gears. For the steel sample in Figure 3, *k*_f_ = 1.1 × 10^7^ N/m. For the resin samples in Figures 4 and 5, *k*_f_ = 6.7 × 10^4^ N/m. Moreover,

$$\begin{array}{c} g_{1}\left(\theta \right)=\frac{\pi \left[-24\pi +4\pi \left(2+\pi \theta -\theta^{2}\right)\cos^{2}\theta +32\sin\theta +\pi \left(4+\pi^{2}\right)\sin^{2}\theta -4\left(\pi -2\theta \right)\cos\theta \left(\pi \sin\theta -4\right)\right]}{{\left(\pi^{2}-8\right)}^{2}} \end{array}$$

$$g_{2}\left(\theta \right)=\frac{\pi \left[-18\pi +\pi^{3}-2\pi \theta^{2}+32\cos\theta +2\pi \left(\theta^{2}-1\right)\cos2\theta +32\theta \sin\theta -4\pi \theta \sin2\theta \right]}{{\left(\pi^{2}-8\right)}^{2}}$$

For other cases of α≠90°, the expressions of stiffness are similar, but we must distinguish the rotation ranges (see Section S4.3 for α = 45°).

When changing θ, we obtain

$$0\leq g_{1}\leq 1$$

$$0.16\leq g_{2}\leq 1$$

Therefore, we have

$$k_{0}\;\leq k_{r}^{\mathrm{comp}}\leq k_{f}+k_{0}$$

$$k_{0}\;\leq k_{r}^{\mathrm{tens}}\leq \frac{\left(k_{0}+k_{f}\right)k_{0}}{\left(k_{0}+0.16k_{f}\right)}$$

If *k*_0_<<*k*_f_, we obtain *k*_0_ ≤$k_{r}^{\mathrm{tens}}$≤6.25*k*_0_ and max($k_{r}^{\mathrm{tens}}$)<<max($k_{r}^{\mathrm{comp}}$). This means that the maximal tunable range of tensile stiffness is of only 6.25 times.

### 4.6 Harmonic Balance Method for Nonlinear Responses

To analytically compute the nonlinear responses, we use a continuous function is used to fit the piecewise function shown in Figure 5b. Assuming *T* = *k*_1_ *φ+k*_2_ *φ* ^2^ *+k*_3_ *φ* ^3^, the fitted relationship between the *T* and the *φ* is shown in Figure S7.

Then, harmonic balance method is employed to solve the torsional frequency response of the mechanical resonator. Without considering damping, the equation of motion is as follows:

$$J\dot{\phi }+k_{1}\phi +k_{2}\phi^{2}+k_{3}\phi^{3}=T_{0}\sin\omega t$$

where *T*_0_sin*ωt* is the external torsional excitation. In calculation, one much transfer the unit of rotation angle from “degree” to “rad”. Assuming the solution of the equation is as follows:

$$\phi =A_{0}+A_{1}\sin\omega t$$

We note that one must include a constant term *A*_0_ in the response because there is a quadratic term in the equation of motion. Substituting it into the equation of motion and ignoring higher‐order harmonics, the following is obtained:

$$\left\{\begin{array}{c} k_{1}A_{0}+k_{2}A_{0}^{2}+k_{3}A_{0}^{3}+\frac{1}{2}k_{2}A_{1}^{2}+\frac{3}{2}k_{3}A_{0}A_{1}^{2}=0\\ \left(-m\omega^{2}+k_{1}\right)A_{1}+2k_{2}A_{0}A_{1}+3k_{2}A_{0}^{2}A_{1}+\frac{3}{4}k_{3}A_{1}^{3}-T_{0}=0 \end{array}\right.$$

Continuation approach is adopted to find the solution of these nonlinear equations. The, one can obtain the bifurcation curves shown in Figure 5.

## Author Contributions

X.F. performed conceptualization, model design, simulations, experiments, writing – original draft and revising, supervision, and funding acquisition. M.Y. performed analytical modeling, simulations of nonlinear dynamics, and experiments. D.Y. performed fabrication, experiments, and editing. L.C. performed writing – review, and editing. X.F. and M.Y. contributed equally to this paper.

## Conflicts of Interest

A patent application has been submitted to China National Intellectual Property Administration. The serial number is 2025119870599. The application date is 2025.12.26. Authors of this paper, Xin Fang, Miao Yu, and Dianlong Yu, are on the patent.

## Supporting information




**Supporting File**: advs74736‐sup‐0001‐SuppMat.pdf.

## Data Availability

The data that supports the findings of this study are available in the supplementary material of this article
