# Supplementary material for: Active Metamaterials with Tunable Shear Nonreciprocity and Nonlinear Dynamics
Source: Adv Sci (Weinh). 2026 Mar 12;13(28):e74736. doi: 10.1002/advs.74736 (PMC13185870; doi:10.1002/advs.74736)
Supplement: Supplementary file 1 — Supporting File: advs74736‐sup‐0001‐SuppMat.pdf. [file ADVS-13-e74736-s001.pdf]

Supporting Information for

Active metamaterials with tunable shear nonreciprocity and nonlinear dynamics

Xin Fang[1,2*†], Miao Yu[1,2†], Dianlong Yu[1,2*], Li Cheng[3*]

[1]National Key Laboratory of Equipment State Sensing and Smart Support, National University of Defense Technology, Changsha, Hunan, China.

[2]College of Intelligent Science and Technology, National University of Defense Technology, Changsha, Hunan, China.

[3] Department of Mechanical Engineering, Hong Kong Polytechnic University, Hong Kong, China

[*]E-mails: xinfangdr@sina.com (X.F.); dianlongyu@vip.sina.com (D.Y); li.cheng@polyu.edu.hk (L.C.).

[†]X.F. and M. Y. contribute equally.

Content in this supplementary file

1. Supplementary figures and corresponding notes 2

2. Vibration test methods 3

3. Equivalent method for the metamaterial based on Taiji gears 4

4. Analytical model for the stiffness of structures based on planetary gears 6

    4.1 Stiffness of a circular ring 6

    4.2 Stiffness of a single planetary gear unit for α=90° 7

    4.3 Stiffness of a single planetary gear unit for α=45° 14

    4.4 Influence of installing angle on the Variable Stiffness 17

5. Torsional stiffness of resonators consisting of planetary gear units 19

6. Tunable nonlinear resonances 21

    6.1 Equations of motion 21

    6.2 Torsional frequency response of mechanical resonator 21

    6.3 Harmonic balance method 22

1. Supplementary figures and corresponding notes

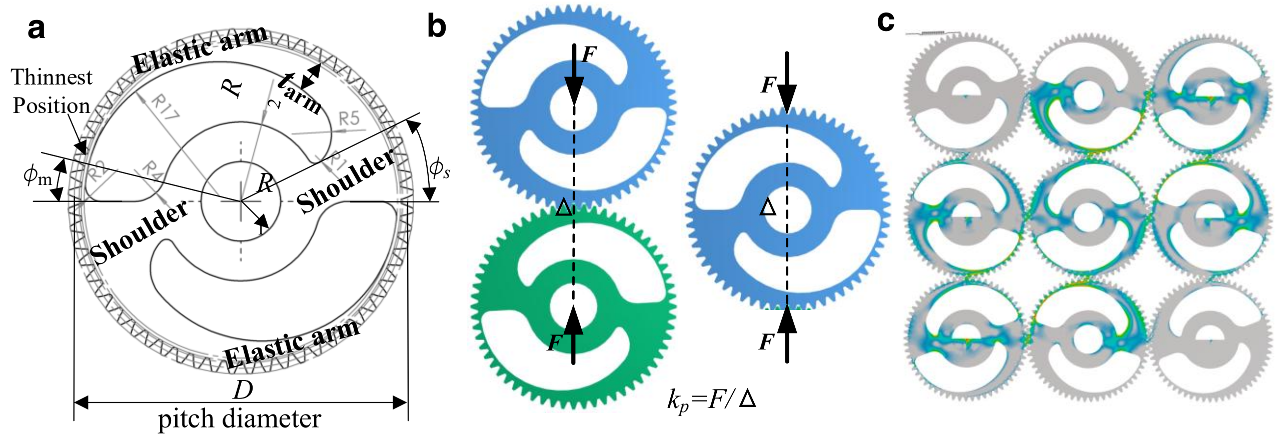

Fig. S1 | Parameters for metamaterials based on Taiji gear. (a) Shape and parameters of a Taiji gear. (b) Compression stiffness k_p between a pair of gears. (c) Shear interlock state of the 3×3 gear array. The whole shape is center-symmetrical. The two solid shoulders are the sections within an azimuthal angle ϕ_s=25°. The radical thickness of the elastic arm t_{arm} varies smoothly with the rotation angle. At ϕ_m=12°, t_{arm} reaches the minimum of 0.424 mm.

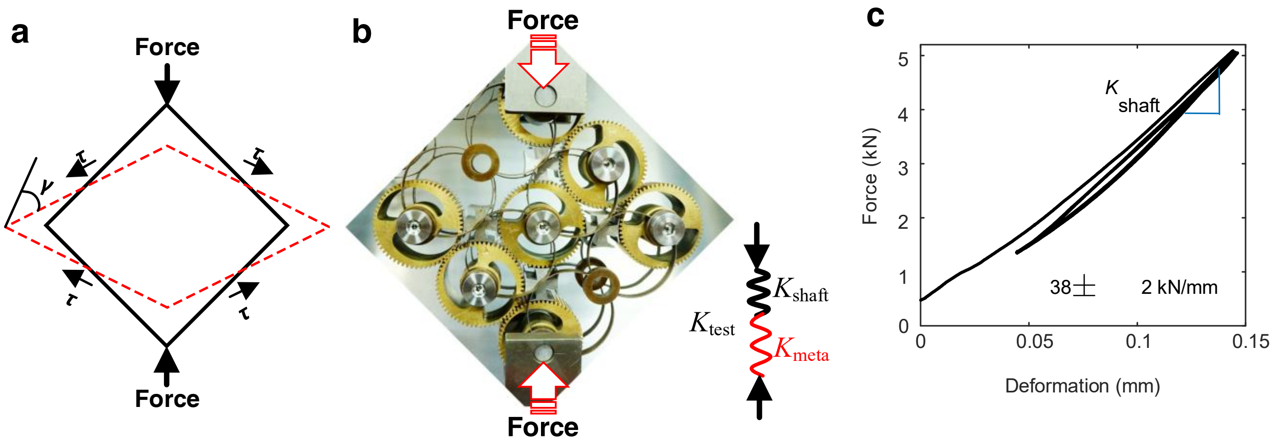

Fig. S2 | Experimental measuring method for the shear stiffness of the metacell based on Taiji gears. (**a**) Experimental illustration for measuring the shear modulus under diagonal compression. (**b**) Picture of the sample. (**c**) Deformation-force relationship of a pair of tandem shafts in (**b**). Their entire stiffness K_{shaft}=38±2 kN/mm is the slope of the curve under larger deformation. Therefore, the stiffness for single shaft is K_{shaft1}=2K_{shaft}.

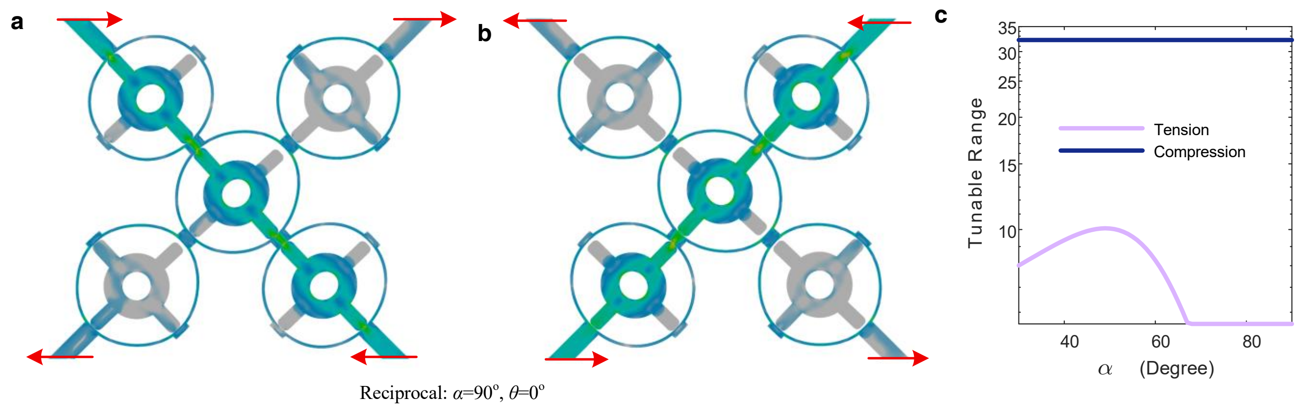

Fig. S3 (a, b) Typical reciprocal deformation modes for α = 90°, θ=0°. (c) Tunable range of the compression and tension stiffness of a planetary unit shown in Fig. 3 in the main text.

2. Vibration test methods

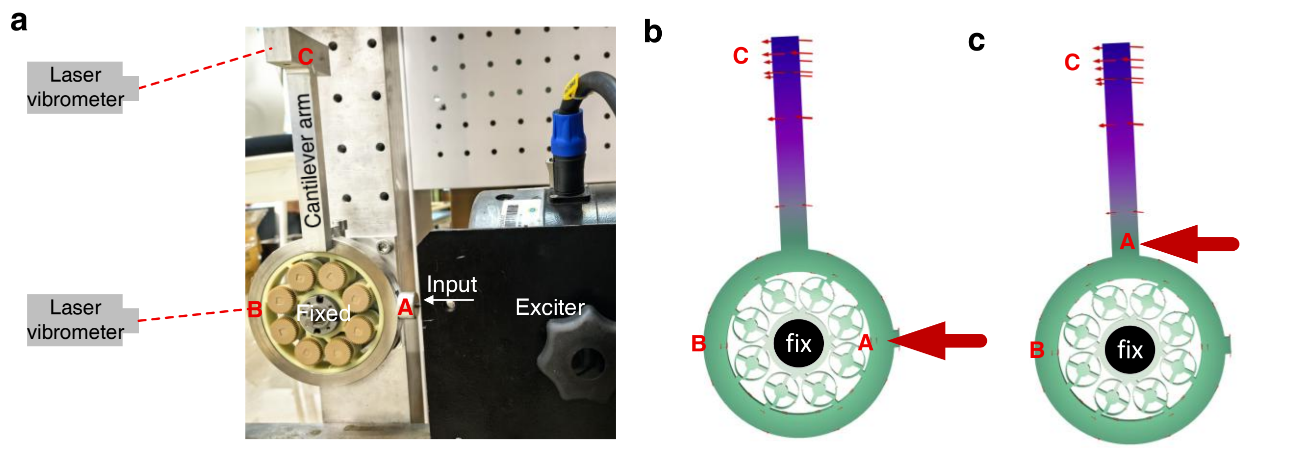

Fig. S4 | Experimental methods for measuring the vibration responses of the metamaterial resonators. (a) Experimental setups. (b,c) Two methods for measuring torsional vibration.

As shown in **Fig. S4**, we fix the inner ring of the entire gear ring and excite the external steel ring to generate vibration. Laser vibrometers are adopted to measure the responses at the excitation and response points. for torsional vibration measurement, a cantilever arm is fixed on the external steel ring to amplify the rotation angle and distinguish the transverse and torsional resonant frequencies. When measuring the pure transverse vibration, no cantilever arm is used. Measuring points A, B, C denote the excitation point, transverse response point, and torsional response point, respectively. We adopt two excitation methods in measuring the torsional responses.

In **Fig. S4**(b), the excitation is applied on 1/4 point of the steel ring. In this case, the excitation

induces transverse vibration on the steel ring, while the asymmetrical cantilever arm generates torsional vibration through the whole structure. Therefore, both large transverse and torsional vibrations are generated on the steel ring. In this case, torsional nonlinear vibration may influence the transverse vibration because of the interaction. This method is only used when measuring the rotational resonant frequency f_{rot}.

In **Fig. S4**(c), the excitation is applied at the root of the cantilever arm (Point A). In this case, the excitation mainly induces torsional vibration. This method is used when measuring the nonlinear responses of the nonreciprocal resonator.

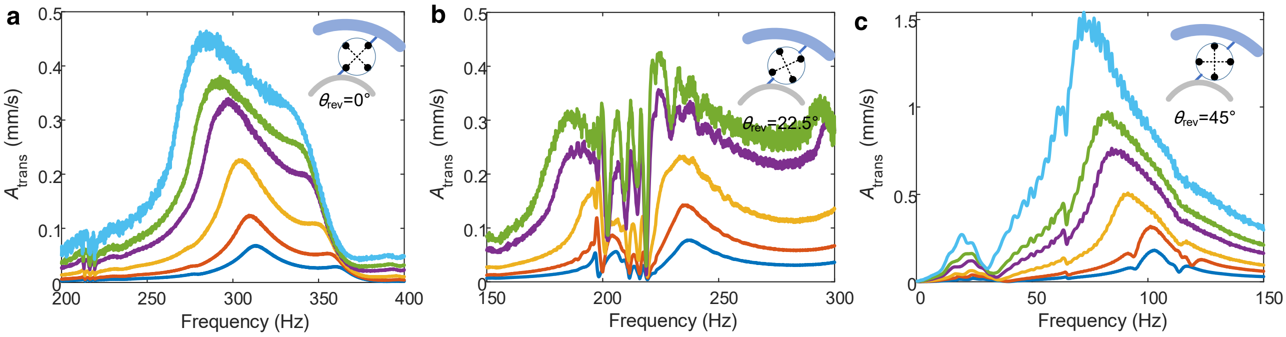

Fig. S5 | Transverse vibration responses when increasing the amplitude. These results are measured using the testing methods in **Fig. S4**(c), corresponding to the response of point B on the steel ring. Responses at point A for indicating the torsional vibration are shown in the main text. (a, b, c) Results for three cases in the pattern α = 90°: θ_{rev}=0°, θ_{rev}=15°, θ_{rev}=45°.

3. Equivalent method for the metamaterial based on Taiji gears

The metacells shown in Fig. 2 in the main text can be equivalent to the discrete model shown in Fig. 2e. The inserted crosses influence not only the modulation of shear modulus G but also the Young's modulus E_y. For the three central gears along y direction, $K_v(\theta)=k_p/2$. The entire stiffnesses in two diagonal directions are:

$$K_{d1}(\theta)=k_p(\theta\text{-}45°)k_{cr}/2[k_p(\theta\text{-}45°)+k_{cr}]$$

$$K_{d2}(\theta)=k_p(\theta+45°)k_{cr}/2[k_p(\theta+45°)+k_{cr}]$$

Here θ denotes the gear rotation angle respective to its coordinate origin; k_{cr} denotes the stiffness of the cross object in the diagonal direction. In the shear stress state, only one arm of the cross is loaded

because the other arm is not in contact with any gear. As shown in **Fig. S6**, the geometrical deformation relationship is

$$(\sqrt{2}a_y - \Delta_g)^2 = (a_x - u)^2 + a_y^2$$

where a_x=2D and a_y=2D denote the lattice constant; u=γa_y is the relative displacement between the two rows of gear centers; γ and Δ_g denote the shear strain and the deformation of the diagonal gears. By neglecting the nonlinear term, one obtains

$$\Delta_g = \sqrt{2}u/2$$

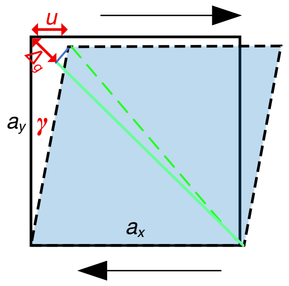

Fig. S6 | Deformation under shear loading.

Therefore, the shear force supported by diagonal gears is

$$F_{x1} = \sqrt{2}K_{d1}\Delta_g / 2 = K_{d1}u/2$$
$$F_{x2} = \sqrt{2}K_{d2}\Delta_g / 2 = K_{d2}u/2$$

When applying shear stress in the positive direction τ^+, the shear force is supported by the diagonal gears connected by crosses 1. Therefore,

$$G_g^+(\theta) = F_{x1}/a_x B\gamma = K_{d1}(\theta)/2B$$

On the contrary,

$$G_g^-(\theta) = F_{x2}/a_x B\gamma = K_{d2}(\theta)/2B$$

Therefore, $G_g^+(\theta)$= $G_g^-(\theta$-90°).

However, when applying the compressive stress σ_y, all gears take part in supporting the stress. In this case, the deformation of the center gear becomes complicated. By using the boundary conditions ε_x=0, γ=0 and v=$\varepsilon_y a_y$, we obtain the equivalent Young's modulus in this case:

$$E_y \approx K_v/B+[K_{d1}(\theta)+ K_{d2}(\theta)]/2B +E_f =G_g^{+}+ G_g^{-}+ K_p/2B+E_f$$

Therefore, the compressive Young's modulus E_y is synchronously tunable.

4. Analytical model for the stiffness of structures based on planetary gears

4.1 Stiffness of a circular ring

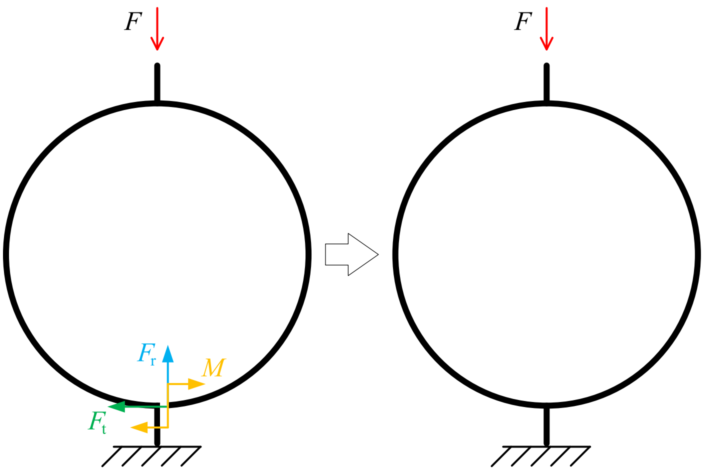

Fig. S7 | Equivalent calculation method for a closed ring

A single planetary gear unit consists of an outer ring, planetary gears, and a sun gear, with two ribs extending from the outer ring serving as force application parts. To solve the equivalent stiffness of a single planetary gear unit under tension and compression, we first consider a planar curved beam with a circumferential angle of 2π, where one end is fixed and the other end is free (the two ends coincide in spatial position but have different boundary conditions), as shown in the left figure of **Fig. S7**. Assuming the curved beam is subjected to a radial force F at the position with the circumferential angle of π, its free end will simultaneously experience a radial force F_r, a tangential force F_t, and a moment M such that the radial displacement, tangential displacement, and rotation angle at the free end are all zero, satisfying [1]:

$$\begin{cases} \dfrac{M\cdot\pi}{r}+F_t\cdot\pi+2F=0 \\ F_r-\dfrac{1}{2}F=0 \\ \dfrac{M\cdot 2\pi}{r}+F_t\cdot 3\pi+2F=0 \end{cases} \tag{1}$$

From Eq. (1), we obtain:

$$\begin{cases} M = -\dfrac{F \cdot r}{\pi} \\ F_r = \dfrac{F}{2} \\ F_t = 0 \end{cases} \tag{2}$$

The radial displacement at the point where force F is applied on the curved beam is:

$$\Delta_0 = \frac{F \cdot \frac{\pi}{2} \cdot r^3 + M \cdot 2r^2 - F_r \cdot \frac{\pi}{2} r^3}{EI} \tag{3}$$

where EI represents the cross-sectional moment of inertia of the curved beam. By substituting Eq. (2) into Eq. (3), we obtain:

$$\Delta_0 = \frac{F \cdot r^3 (\pi^2 - 8)}{4EI\pi} \tag{4}$$

The stiffness at the point where force F is applied on the curved beam is:

$$k_0 = F / \Delta_0 = \frac{4EI\pi}{r^3 (\pi^2 - 8)} \tag{5}$$

The above calculation process can represent the solution for the equivalent stiffness of a closed ring. Subsequent calculations are also based on this method.

4.2 Stiffness of a single planetary gear unit for α=90°

Next, consider the deformation at any position on the ring when subjected to force F, as shown in **Fig. S8**. When the ring deforms under external force, the radial deformation Δ_1 at any position θ and the radial deformation Δ_2 at its diametrically opposite position can be obtained by Eq. (6) and Eq. (7):

$$\Delta_1 = \frac{F \cdot r^3}{EI} \cdot (\frac{\pi - \theta}{2} \cos\theta + \frac{1}{2} \sin\theta) + \frac{M \cdot r^2}{EI} (1 - \cos\theta) + \frac{F_r \cdot r^3}{EI} (-\frac{\pi - \theta}{2} \cos\theta - \frac{1}{2} \sin\theta) \tag{6}$$

$$\Delta_2 = \frac{F \cdot r^3}{EI} \cdot (-\frac{\pi}{2} \cos\theta) + \frac{M \cdot r^2}{EI} (1 - \cos\theta) + \frac{F_r \cdot r^3}{EI} \cdot (\frac{2\pi - \theta}{2} \cos\theta) \tag{7}$$

Substituting Eq. (2) and simplifying yields:

$$\Delta_1 = \frac{F \cdot r^3}{EI} \cdot (\frac{\pi - \theta}{4} \cos\theta + \frac{1}{4} \sin\theta - \frac{1}{\pi} - \frac{\cos\theta}{\pi}) \tag{8}$$

$$\Delta_2 = \frac{F \cdot r^3}{EI} \cdot (-\frac{\theta}{4} \cos\theta + \frac{1}{4} \sin\theta - \frac{1}{\pi} + \frac{\cos\theta}{\pi}) \tag{9}$$

Here, r is the centroid radius of the ring, E is the Young's modulus of the ring material, and I is the moment of inertia of the ring cross-section, calculated as I=12bh^3, where b is the width of the ring cross-section and h is the thickness of the ring cross-section. The sign of the deformation (positive or negative) indicates the direction of deformation: positive represents the centripetal direction, and negative represents the centrifugal direction. From Eq. (8) and Eq. (9), the total deformation Δ at position θ and its diametrically opposite position is:

$$\Delta = \Delta_1 + \Delta_2 = \frac{F \cdot r^3}{EI} \cdot \left(\frac{\pi - 2\theta}{4}\cos\theta + \frac{1}{2}\sin\theta - \frac{2}{\pi}\right) \tag{10}$$

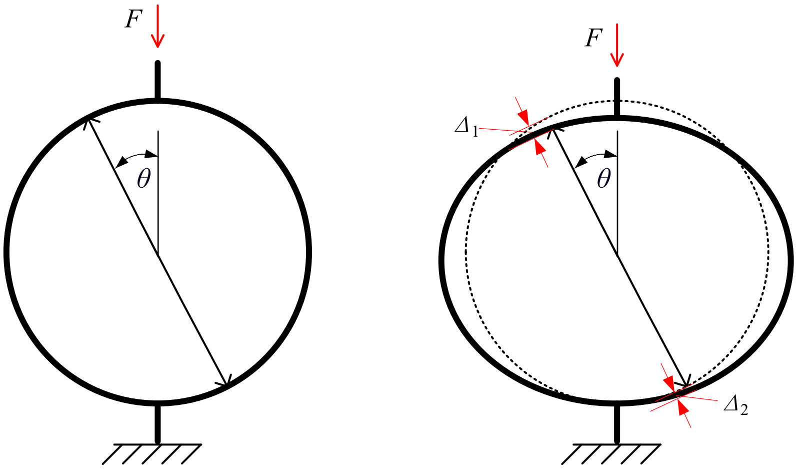

Fig. S8 | Deformation solution for a closed ring

A positive Δ indicates a decrease in the relative distance between the two diametrically opposite points on the ring, while a negative Δ indicates an increase. If there is a fulcrum (planetary gear and sun gear) at position θ, when the relative distance of the ring decreases it will compresses the fulcrum and the fulcrum can provide a reaction force. While when the relative distance increases, the ring separates from the fulcrum, and the fulcrum can not provides force.

Define the deformation coefficient μ=(ΔEI)/(Fr^3). The value of μ at different θ is shown in **Fig. S9**. The sign of μ indicates the ring's deformation behavior at different positions: when $\mu > 0$, the radial relative distance at θ and its diametrically opposite position changes in the same direction as the applied force; when $\mu < 0$, it changes in the opposite direction. According to convention, centripetal force is positive and centrifugal force is negative, while a decrease in radial distance is considered positive and an increase negative. When fulcrums are placed at θ and its opposite point,

their effectiveness depends on the sign of μ: a fulcrum functions only if the radial distance at that position decreases. Thus, under centripetal force, positions with $\mu > 0$ provide effective fulcrums, whereas under centrifugal force, positions with $\mu < 0$ are effective.

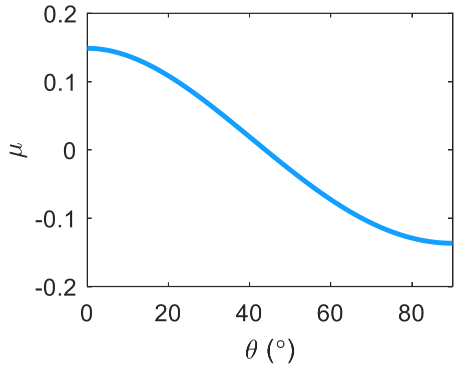

Fig. S9 | Value of deformation coefficient μ at different θ

Now consider two pairs of fulcrums inside the ring, located at θ and at an angle α from θ. The stiffness of the fulcrums is k_f, as shown in **Fig. S10**.

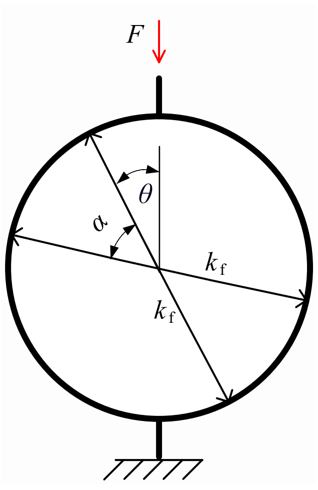

Fig. S10 | Force analysis of a closed ring with internal fulcrums

Define the functions:

$$f_C(\theta) = \begin{cases} 1 & , \frac{\pi - 2\theta}{4}\cos\theta + \frac{1}{2}\sin\theta - \frac{2}{\pi} > 0 \\ 0 & , \frac{\pi - 2\theta}{4}\cos\theta + \frac{1}{2}\sin\theta - \frac{2}{\pi} \le 0 \end{cases} \tag{11}$$

$$f_T(\theta)=\begin{cases}1 & ,\frac{\pi-2\theta}{4}\cos\theta+\frac{1}{2}\sin\theta-\frac{2}{\pi}<0\\ 0 & ,\frac{\pi-2\theta}{4}\cos\theta+\frac{1}{2}\sin\theta-\frac{2}{\pi}\geq 0\end{cases} \tag{12}$$

Then, based on the force balance equations, the force balance equation for the ring under compression is:

$$\begin{cases}\frac{M\cdot\pi}{r}+F_t\cdot\pi+F+F_1\cdot f_C(\theta)+F_2\cdot f_C(\theta+\alpha)=0\\ F_r\cdot\pi-\frac{\pi}{2}F+\frac{\pi}{2}\cos\theta\cdot F_1\cdot f_C(\theta)+\frac{\pi}{2}\cos(\theta+\alpha)\cdot F_2\cdot f_C(\theta+\alpha)=0\\ \frac{M\cdot 2\pi}{r}+F_t\cdot 3\pi+2F+F_1\cdot(2+\frac{\pi}{2}\sin\theta)\cdot f_C(\theta)\\ +F_2\cdot(2+\frac{\pi}{2}\sin(\theta+\alpha))\cdot f_C(\theta+\alpha)=0\\ (\frac{M\cdot 2}{r}+F_r\cdot\frac{\pi}{2}\cos\theta+F_t\cdot(2+\frac{\pi}{2}\sin\theta)+F\cdot(-\frac{\theta}{2}\cos\theta+\frac{1}{2}\sin\theta)+F_1\cdot\frac{\pi}{2}\\ +F_2\cdot\frac{1}{2}\cdot(\frac{\pi-\alpha}{2}\cos\alpha+\frac{1}{2}\sin\alpha)\cdot f_C(\theta+\alpha))\cdot f_C(\theta)=(-\frac{F_1\cdot EI}{k_f r^3})\cdot f_C(\theta)\\ (\frac{M\cdot 2}{r}+F_r\cdot\frac{\pi}{2}\cos(\theta+\alpha)+F_t\cdot(2+\frac{\pi}{2}\sin(\theta+\alpha))+F\cdot(-\frac{(\theta+\alpha)}{2}\cos(\theta+\alpha)+\frac{1}{2}\sin(\theta+\alpha))\\ +F_2\cdot\frac{\pi}{2}+F_1\cdot\frac{1}{2}\cdot(\frac{\pi-\alpha}{2}\cos\alpha+\frac{1}{2}\sin\alpha)\cdot f_C(\theta))\cdot f_C(\theta+\alpha)=(-\frac{F_2\cdot EI}{k_f r^3})\cdot f_C(\theta+\alpha)\end{cases} \tag{13}$$

The force balance equation for the ring under tension is:

$$\begin{cases}\frac{M\cdot\pi}{r}+F_t\cdot\pi+F+F_1\cdot f_T(\theta)+F_2\cdot f_T(\theta+\alpha)=0\\ F_r\cdot\pi-\frac{\pi}{2}F+\frac{\pi}{2}\cos\varphi\cdot F_1\cdot f_T(\theta)+\frac{\pi}{2}\cos(\theta+\alpha)\cdot F_2\cdot f_T(\theta+\alpha)=0\\ \frac{M\cdot 2\pi}{r}+F_t\cdot 3\pi+2F+F_1\cdot(2+\frac{\pi}{2}\sin\theta)\cdot f_T(\theta)\\ +F_2\cdot(2+\frac{\pi}{2}\sin(\theta+\alpha))\cdot f(\theta+\alpha)=0\\ (\frac{M\cdot 2}{r}+F_r\cdot\frac{\pi}{2}\cos\theta+F_t\cdot(2+\frac{\pi}{2}\sin\theta)+F\cdot(-\frac{\theta}{2}\cos\theta+\frac{1}{2}\sin\theta)+F_1\cdot\frac{\pi}{2}\\ +F_2\cdot\frac{1}{2}\cdot(\frac{\pi-\alpha}{2}\cos(\alpha)+\frac{1}{2}\sin(\alpha))\cdot f_T(\theta+\alpha))\cdot f_T(\theta)=(-\frac{F_1\cdot EI}{k_f r^3})\cdot f(\theta)\\ (\frac{M\cdot 2}{r}+F_r\cdot\frac{\pi}{2}\cos(\theta+\alpha)+F_t\cdot(2+\frac{\pi}{2}\sin(\theta+\alpha))+F\cdot(-\frac{(\theta+\alpha)}{2}\cos(\theta+\alpha)+\frac{1}{2}\sin(\theta+\alpha))\\ +F_2\cdot\frac{\pi}{2}+F_1\cdot\frac{1}{2}\cdot(\frac{\pi-\alpha}{2}\cos(\alpha)+\frac{1}{2}\sin(\alpha))\cdot f_T(\theta))\cdot f_T(\theta+\alpha)=(-\frac{F_2\cdot EI}{k_f r^3})\cdot f_T(\theta+\alpha)\end{cases} \tag{14}$$

Specifically, when $\alpha = 90°$, the internal fulcrum forces under compression and tension are shown in **Fig. S11**.

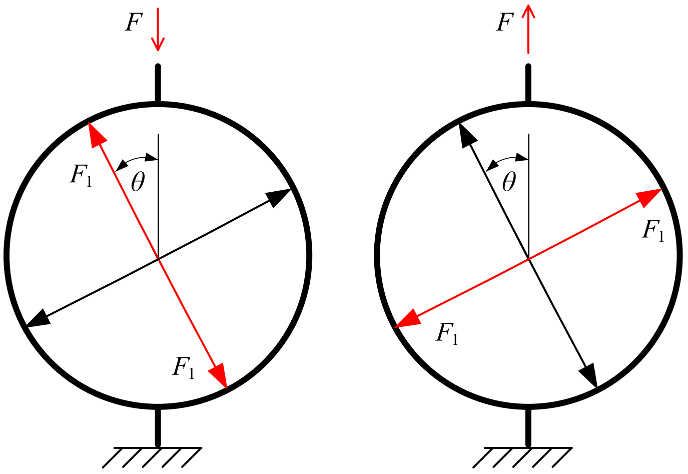

Fig. S11 | Internal fulcrum forces when *α* = 90° under compression and tension

From **Fig. S11**, the force balance equation for the ring under compression is:

$$\begin{cases} \dfrac{M\cdot\pi}{r} + F_t\cdot\pi + F + F_1 = 0 \\ F_r\cdot\pi - \dfrac{\pi}{2}F + \dfrac{\pi}{2}\cos\theta\cdot F_1 = 0 \\ \dfrac{M\cdot 2\pi}{r} + F_t\cdot 3\pi + 2F + F_1\cdot(2+\dfrac{\pi}{2}\sin\theta) = 0 \\ \dfrac{M\cdot 2}{r} + F_r\cdot\dfrac{\pi}{2}\cos\theta + F_t\cdot(2+\dfrac{\pi}{2}\sin\theta) + F\cdot(-\dfrac{\theta}{2}\cos\theta + \dfrac{1}{2}\sin\theta) + F_1\cdot\dfrac{\pi}{2} = -\dfrac{F_1\cdot EI}{k_f r^3} \end{cases} \tag{15}$$

From the above equation, the relationships between M, F_r, F_t, F_1 and F can be obtained:

$$\begin{cases} M = -Fr\dfrac{(-2k_f\pi r^3\cos^2\theta) + k_f r^3(\pi-2\theta)\cos\theta(-2+\pi\sin\theta) + 4(2EI + k_s\pi r^3 - 3k_f r^3\sin\theta)}{8EI\pi + 2k_f(-8+\pi^2)r^3} \\ F_r = -F\dfrac{-4EI\pi + 8k_f r^3 - 2k_f\pi r^3 + 2k_f\pi r^3\theta\cos^2\theta + k_f\pi^2 r^3\sin^2\theta - 2k_f r^3\cos\theta(-4+\pi\sin\theta)}{8EI\pi + 2k_f(-8+\pi^2)r^3} \\ F_t = F\dfrac{k_f r^3\sin\theta[-8+\pi(\pi-2\theta)\cos\theta + 2\pi\sin\theta]}{8EI\pi + 2k_f(-8+\pi^2)r^3} \\ F_1 = F\dfrac{k_f r^3[-8+\pi(\pi-2\theta)\cos\theta + 2\pi\sin\theta]}{4EI\pi + k_f(-8+\pi^2)r^3} \end{cases} \tag{16}$$

The deformation at the point where force F is applied can then be obtained:

$$\delta = \frac{M\cdot 2r^2 + F\cdot\dfrac{\pi}{2}r^3 + F_t\cdot 2r^3 - F_r\cdot\dfrac{\pi}{2}r^3 + F_1\cdot r^3\cdot(-\dfrac{\theta}{2}\cos\theta + \dfrac{1}{2}\sin\theta)}{EI} \tag{17}$$

Substituting and simplifying gives:

$$\delta = \frac{Fr^3[4EI(-8+\pi^2) - 24k_f\pi r^3 + 4k_f\pi r^3(2+\pi\theta-\theta^2)\cos^2\theta + 32k_f r^3\sin\theta + k_f\pi(4+\pi^2)r^3\sin^2\theta - 4k_f r^3(\pi-2\theta)\cos\theta(\pi\sin\theta-4)]}{4EI[4EI\pi + k_f(\pi^2-8)r^3]} \tag{18}$$

The equivalent stiffness of the ring is then:

$$\begin{aligned} k_r = F/\delta &= \frac{4EI[4EI\pi + k_f(\pi^2-8)r^3]}{r^3[4EI(-8+\pi^2) - 24k_f\pi r^3 + 4k_f\pi r^3(2+\pi\theta-\theta^2)\cos^2\theta + 32k_f r^3\sin\theta + k_f\pi(4+\pi^2)r^3\sin^2\theta - 4k_f r^3(\pi-2\theta)\cos\theta(\pi\sin\theta-4)]} \\ &= \frac{\frac{4EI}{r^3}[\frac{4EI\pi}{k_f r^3} + (\pi^2-8)]}{[\frac{4EI(\pi^2-8)}{k_f r^3} - 24\pi + 4\pi(2+\pi\theta-\theta^2)\cos^2\theta + 32\sin\theta + \pi(4+\pi^2)\sin^2\theta - 4(\pi-2\theta)\cos\theta(\pi\sin\theta-4)]} \\ &= \frac{(k_0+k_f)k_0}{k_0 + k_f\frac{\pi[-24\pi + 4\pi(2+\pi\theta-\theta^2)\cos^2\theta + 32\sin\theta + \pi(4+\pi^2)\sin^2\theta - 4(\pi-2\theta)\cos\theta(\pi\sin\theta-4)]}{(\pi^2-8)^2}} \end{aligned} \tag{19}$$

Let

$$g_1(\theta) = \frac{\pi[-24\pi + 4\pi(2+\pi\theta-\theta^2)\cos^2\theta + 32\sin\theta + \pi(4+\pi^2)\sin^2\theta - 4(\pi-2\theta)\cos\theta(\pi\sin\theta-4)]}{(\pi^2-8)^2} \tag{20}$$

Then the equivalent compressive stiffness of the ring is:

$$k_r = \frac{(k_0+k_f)k_0}{k_0 + k_f g_1(\theta)} \tag{21}$$

Similarly, the force balance equation for the ring under tension is:

$$\begin{cases} \frac{M\cdot\pi}{r} + F_t\cdot\pi + F + F_1 = 0 \\ F_r\cdot\pi - \frac{\pi}{2}F - \frac{\pi}{2}\sin\theta\cdot F_1 = 0 \\ \frac{M\cdot 2\pi}{r} + F_t\cdot 3\pi + 2F + F_1\cdot(2-\frac{\pi}{2}\cos\theta) = 0 \\ \frac{M\cdot 2}{r} - F_r\cdot\frac{\pi}{2}\sin\theta + F_t\cdot(2-\frac{\pi}{2}\cos\theta) + F\cdot(\frac{\theta+\frac{\pi}{2}}{2}\sin\theta - \frac{1}{2}\cos\theta) + F_1\cdot\frac{\pi}{2} = -\frac{F_1\cdot EI}{k_f r^3} \end{cases} \tag{22}$$

From the above equation, the relationships between M, F_r, F_t, F_1 and F can be obtained:

$$\begin{cases} M = -Fr\dfrac{4EI + 2k_f\pi r^3 - 2k_f r^3\theta\sin\theta - k_f\pi r^3\sin^2\theta + k_f r^3\cos\theta(-6 + \pi\theta\sin\theta)}{4EI\pi + k_f(-8+\pi^2)r^3} \\ F_r = F\dfrac{4EI\pi - 8k_f r^3 + k_f\pi^2 r^3 - k_f\pi r^3\theta + k_f\pi r^3\theta\cos 2\theta + 8k_f r^3\sin\theta - k_f\pi r^3\sin 2\theta}{8EI\pi + 2k_f(-8+\pi^2)r^3} \\ F_t = F\dfrac{k_f r^3\cos\theta[-4 + \pi\cos\theta + \pi\theta\sin\theta]}{4EI\pi + k_f(-8+\pi^2)r^3} \\ F_1 = -F\dfrac{2k_f r^3(-4 + \pi\cos\theta + \pi\theta\sin\theta)}{4EI\pi + k_f(-8+\pi^2)r^3} \end{cases} \tag{23}$$

The deformation at the point where force F is applied is:

$$\delta = \frac{M\cdot 2r^2 + F\cdot\frac{\pi}{2}r^3 + F_t\cdot 2r^3 \text{-} F_r\cdot\frac{\pi}{2}r^3 + F_1\cdot r^3\cdot\left(\frac{\theta+\frac{\pi}{2}}{2}\sin\theta - \frac{1}{2}\cos\theta\right)}{EI} \tag{24}$$

Substituting and simplifying gives:

$$\delta = \frac{\begin{array}{c} Fr^3[4EI(-8+\pi^2) - 18k_f\pi r^3 + k_f\pi^3 r^3 - 2k_f\pi r^3\theta^2 + 32k_f r^2\cos\theta \\ + 2k_f\pi r^3(\theta^2-1)\cos 2\theta + 32k_f r^3\theta\sin\theta - 4k_f\pi r^3\theta\sin 2\theta] \end{array}}{4EI[4EI\pi + k_f(\pi^2-8)r^3]} \tag{25}$$

Similarly, the equivalent tensile stiffness of the ring is:

$$\begin{aligned} k_r = F/\delta &= \frac{4EI[4EI\pi + k_f(\pi^2-8)r^3]}{\begin{array}{c} r^3[4EI(-8+\pi^2) - 18k_f\pi r^3 + k_f\pi^3 r^3 - 2k_f\pi r^3\theta^2 + 32k_f r^3\cos\theta \\ + 2k_f\pi r^3(\theta^2-1)\cos 2\theta + 32k_f r^3\theta\sin\theta - 4k_f\pi r^3\theta\sin 2\theta] \end{array}} \\ &= \frac{\frac{4EI}{r^3}[\frac{4EI\pi}{k_f r^3} + (\pi^2-8)]}{[\frac{4EI(\pi^2-8)}{k_f r^3} - 18\pi + \pi^3 - 2\pi\theta^2 + 32\cos\theta + 2\pi(\theta^2-1)\cos 2\theta + 32\theta\sin\theta - 4\pi\theta\sin 2\theta]} \\ &= \frac{(k_0+k_f)k_0}{k_0 + k_f\frac{\pi[-18\pi + \pi^3 - 2\pi\theta^2 + 32\cos\theta + 2\pi(\theta^2-1)\cos 2\theta + 32\theta\sin\theta - 4\pi\theta\sin 2\theta]}{(\pi^2-8)^2}} \end{aligned} \tag{26}$$

Let

$$g_2(\theta) = \frac{\pi[-18\pi + \pi^3 - 2\pi\theta^2 + 32\cos\theta + 2\pi(\theta^2-1)\cos 2\theta + 32\theta\sin\theta - 4\pi\theta\sin 2\theta]}{(\pi^2-8)^2} \tag{27}$$

Then the equivalent tensile stiffness of the ring is:

$$k_r = \frac{(k_0+k_f)k_0}{k_0 + k_f g_2(\theta)} \tag{28}$$

Equations (21) and (28) can be used to calculate the equivalent stiffness of the planetary gear unit under tension and compression. With r=12.8 mm, b=20 mm, h=0.65 mm, E=2 GPa, k_f=6.7×10^4 N/m, the equivalent stiffness of the planetary gear unit under tension and compression for different values of θ, obtained from Eqs. (21) and (28), is shown in **Fig. S12**.

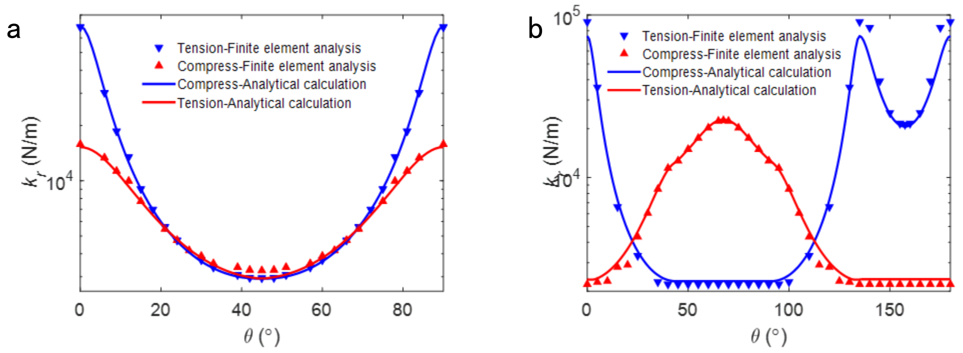

Fig. S12 | Equivalent stiffness results of a single planetary gear unit. (a) α=90° (b) α=45°.

Finite element analysis is performed on the actual structure to verify the accuracy of the proposed calculation method. As shown in **Fig. S12**a, the equivalent stiffness values obtained from FEA across different fulcrum angles θ are in close agreement with those from theoretical calculations. This validates the predictive accuracy of our method for the ring structure's equivalent stiffness and establishes a solid basis for the subsequent development and optimization of the gear variable stiffness structure.

4.3 Stiffness of a single planetary gear unit for α=45°

The aforementioned analysis is conducted for the case of α = 90°. It should be noted that the planetary gear unit exhibits distinct stiffness characteristics when α = 45°. First, its equivalent tensile and compressive stiffness is calculated. The calculation method is the same as above, but the force balance equations will be different. The force balance equations for a single planetary gear unit are as follows:

When 0 °< θ < 45°, under compression:

$$\begin{cases} \frac{M \cdot \pi}{r} + F_t \cdot \pi + F + F_1 = 0 \\ F_r \cdot \pi - \frac{\pi}{2} F + \frac{\pi}{2} \cos\theta \cdot F_1 = 0 \\ \frac{M \cdot 2\pi}{r} + F_t \cdot 3\pi + 2F + F_1 \cdot (2 + \frac{\pi}{2} \sin\theta) = 0 \\ \frac{M \cdot 2}{r} + F_r \cdot \frac{\pi}{2} \cos\theta + F_t \cdot (2 + \frac{\pi}{2} \sin\theta) + F \cdot (-\frac{\theta}{2} \cos\theta + \frac{1}{2} \sin\theta) + F_1 \cdot \frac{\pi}{2} = -\frac{F_1 \cdot EI}{k_s r^3} \end{cases} \tag{29}$$

Under tension:

$$\begin{cases} \frac{M \cdot \pi}{r} + F_t \pi + F + F_1 = 0 \\ F_r \cdot \pi - \frac{\pi}{2} F + \frac{\pi}{2} \cos\theta \cdot F_1 \cdot = 0 \\ \frac{M \cdot 2\pi}{r} + F_t \cdot 3\pi + 2F \cdot r^3 + F_1 \cdot (2 + \frac{\pi}{2} \sin(\theta + \frac{\pi}{4})) = 0 \\ \frac{M \cdot 2}{r} + F_r \cdot \frac{\pi}{2} \cos(\theta + \frac{\pi}{4}) + F_t \cdot (2 + \frac{\pi}{2} \sin(\theta + \frac{\pi}{4})) \\ + F \cdot (-\frac{(\theta + \frac{\pi}{4})}{2} \cos(\theta + \frac{\pi}{4}) + \frac{1}{2} \sin(\theta + \frac{\pi}{4})) + F_1 \cdot \frac{\pi}{2} = -\frac{F_1 \cdot EI}{k_s r^3} \end{cases} \tag{30}$$

When 45°<θ<90°, under compression:

$$\begin{cases} \frac{M \cdot \pi}{r} + F_t \cdot \pi + 2F = 0 \\ F_r - \frac{1}{2} F = 0 \\ \frac{M \cdot 2\pi}{r} + F_t \cdot 3\pi + 2F = 0 \end{cases} \tag{31}$$

Under tension:

$$\begin{cases} \dfrac{M \cdot \pi}{r} + F_t \cdot \pi + F + F_1 + F_2 = 0 \\ F_r \cdot \pi - \dfrac{\pi}{2}F + \dfrac{\pi}{2}\cos\theta \cdot F_1 + \dfrac{\pi}{2}\cos(\theta + \dfrac{\pi}{4}) \cdot F_2 = 0 \\ \dfrac{M \cdot 2\pi}{r} + F_t \cdot 3\pi + 2F + F_1 \cdot (2 + \dfrac{\pi}{2}\sin\theta) + F_2 \cdot (2 + \dfrac{\pi}{2}\sin(\theta + \dfrac{\pi}{4})) = 0 \\ \dfrac{M \cdot 2}{r} + F_r \cdot \dfrac{\pi}{2}\cos\theta + F_t \cdot (2 + \dfrac{\pi}{2}\sin\theta) + F \cdot (-\dfrac{\theta}{2}\cos\theta + \dfrac{1}{2}\sin\theta) \\ + F_1 \cdot \dfrac{\pi}{2} + F_2 \cdot \dfrac{\sqrt{2}}{2}(\dfrac{3\pi}{8} + \dfrac{1}{2}) = -\dfrac{F_1 \cdot EI}{k_s r^3} \\ \dfrac{M \cdot 2}{r} + F_r \cdot \dfrac{\pi}{2}\cos(\theta + \dfrac{\pi}{4}) + F_t \cdot (2 + \dfrac{\pi}{2}\sin(\theta + \dfrac{\pi}{4})) \\ + F \cdot (-\dfrac{(\theta + \dfrac{\pi}{4})}{2}\cos(\theta + \dfrac{\pi}{4}) + \dfrac{1}{2}\sin(\theta + \dfrac{\pi}{4})) + F_1 \cdot \dfrac{\sqrt{2}}{2}(\dfrac{3\pi}{8} + \dfrac{1}{2}) + F_2 \cdot \dfrac{\pi}{2} = -\dfrac{F_2 \cdot EI}{k_s r^3} \end{cases} \quad (32)$$

When 90°<θ<135°, under compression:

$$\begin{cases} \dfrac{M \cdot \pi}{r} + F_t \pi + F + F_1 = 0 \\ F_r \cdot \pi - \dfrac{\pi}{2}F + \dfrac{\pi}{2}\cos(\dfrac{3\pi}{4} - \theta) \cdot F_1 = 0 \\ \dfrac{M \cdot 2\pi}{r} + F_t \cdot 3\pi + 2F + F_1 \cdot (2 + \dfrac{\pi}{2}\sin(\dfrac{3\pi}{4} - \theta)) = 0 \\ \dfrac{M \cdot 2}{r} + F_r \cdot \dfrac{\pi}{2}\cos(\dfrac{3\pi}{4} - \theta) + F_t \cdot (2 + \dfrac{\pi}{2}\sin(\dfrac{3\pi}{4} - \theta)) + F \cdot (-\dfrac{\dfrac{3\pi}{4} - \theta}{2}\cos(\dfrac{3\pi}{4} - \theta) \\ + \dfrac{1}{2}\sin(\dfrac{3\pi}{4} - \theta)) + F_1 \cdot \dfrac{\pi}{2} = -\dfrac{F_1 \cdot EI}{k_s r^3} \end{cases} \quad (33)$$

Under tension:

$$\begin{cases} \dfrac{M \cdot \pi}{r} + F_t \pi + F + F_1 = 0 \\ F_r \cdot \pi - \dfrac{\pi}{2}F + \dfrac{\pi}{2}\cos(\pi - \theta) \cdot F_1 = 0 \\ \dfrac{M \cdot 2\pi}{r} + F_t \cdot 3\pi + 2F + F_1 \cdot (2 + \dfrac{\pi}{2}\sin(\pi - \theta)) = 0 \\ \dfrac{M \cdot 2}{r} + F_r \cdot \dfrac{\pi}{2}\cos(\pi - \theta) + F_t \cdot (2 + \dfrac{\pi}{2}\sin(\pi - \theta)) + F \cdot [-\dfrac{\pi - \theta}{2}\cos(\pi - \theta) \\ + \dfrac{1}{2}\sin(\pi - \theta)] + F_1 \cdot \dfrac{\pi}{2} = -\dfrac{F_1 \cdot EI}{k_s r^3} \end{cases} \quad (34)$$

When 135°<θ<180°, under compression:

$$\begin{cases} \frac{M\cdot\pi}{r}+F_t\cdot\pi+F+F_1+F_2=0 \\ F_r\cdot\pi-\frac{\pi}{2}F+\frac{\pi}{2}\cos(\theta-\frac{3\pi}{4})\cdot F_1+\frac{\pi}{2}\cos\theta\cdot F_2=0 \\ \frac{M\cdot 2\pi}{r}+F_t\cdot 3\pi+2F+F_1\cdot(2+\frac{\pi}{2}\sin(\theta-\frac{3\pi}{4}))+F_2\cdot(2+\frac{\pi}{2}\sin\theta)=0 \\ \frac{M\cdot 2}{r}+F_r\cdot\frac{\pi}{2}\cos(\theta-\frac{3\pi}{4})+F_t\cdot(2+\frac{\pi}{2}\sin(\theta-\frac{3\pi}{4}))+F\cdot(-\frac{\theta}{2}\cos(\theta-\frac{3\pi}{4})+\frac{1}{2}\sin(\theta-\frac{3\pi}{4})) \\ +F_1\cdot\frac{\pi}{2}+F_2\cdot\frac{\sqrt{2}}{2}(-\frac{\pi}{8}+\frac{1}{2})=-\frac{F_1\cdot EI}{k_s r^3} \\ \frac{M\cdot 2}{r}+F_r\cdot\frac{\pi}{2}\cos\theta+F_t\cdot(2+\frac{\pi}{2}\sin\theta) \\ +F\cdot(-\frac{\theta}{2}\cos\theta+\frac{1}{2}\sin\theta)+F_1\cdot\frac{\sqrt{2}}{2}(-\frac{\pi}{8}+\frac{1}{2})+F_2\cdot\frac{\pi}{2}=-\frac{F_2\cdot EI}{k_s r^3} \end{cases} \tag{36}$$

Under tension:

$$\begin{cases} \frac{M\cdot\pi}{r}+F_t\cdot\pi+2F=0 \\ F_r-\frac{1}{2}F=0 \\ \frac{M\cdot 2\pi}{r}+F_t\cdot 3\pi+2F=0 \end{cases} \tag{37}$$

Based on the force balance equations, the equivalent tension and compression stiffness under different θ of a single planetary gear unit can be obtained can be derived using the same methodology as presented in Eqs. 17-21. Under identical structural and material parameters, the equivalent stiffness for α=45° is shown in **Fig. S12**b. Similarly, finite element analysis is performed on the actual structure to calculate its equivalent stiffness for comparison with the theoretical calculations. The results from both methods are also in good agreement, further validating the correctness of the theoretical calculations.

4.4 Influence of installing angle on the Variable Stiffness

A comparison of **Fig. S12**a and **Fig. S12**b reveals that the minimum tensile and compressive stiffness (no fulcrum) remains largely unaffected by α. While the maximum compressive stiffness (θ=0°) also shows minimal dependence on α, the maximum tensile stiffness at α = 45° exhibits a significant increase compared to that at α = 90°. Moreover, the support position corresponding to the maximum tensile stiffness is also different. The maximum tensile stiffness corresponds to two

fulcrum methods and depends on *α*, as shown in **Fig. S13**. We define Change ratio *CR*= Max tension stiffness / Min tension stiffness and obtained *CR* under different *α* through calculation method mentioned in Section 4.2 and 4.3, as shown in **Fig. S13**c. When 0°< *α* <67°, the fulcrum methods of max tension stiffness is **Fig. S13**b, and when *α* >67° the fulcrum methods of max tension stiffness is **Fig. S13**a. The max tension stiffness can be designed according to actual engineering needs under the condition that the fulcrum stiffness has been determined.

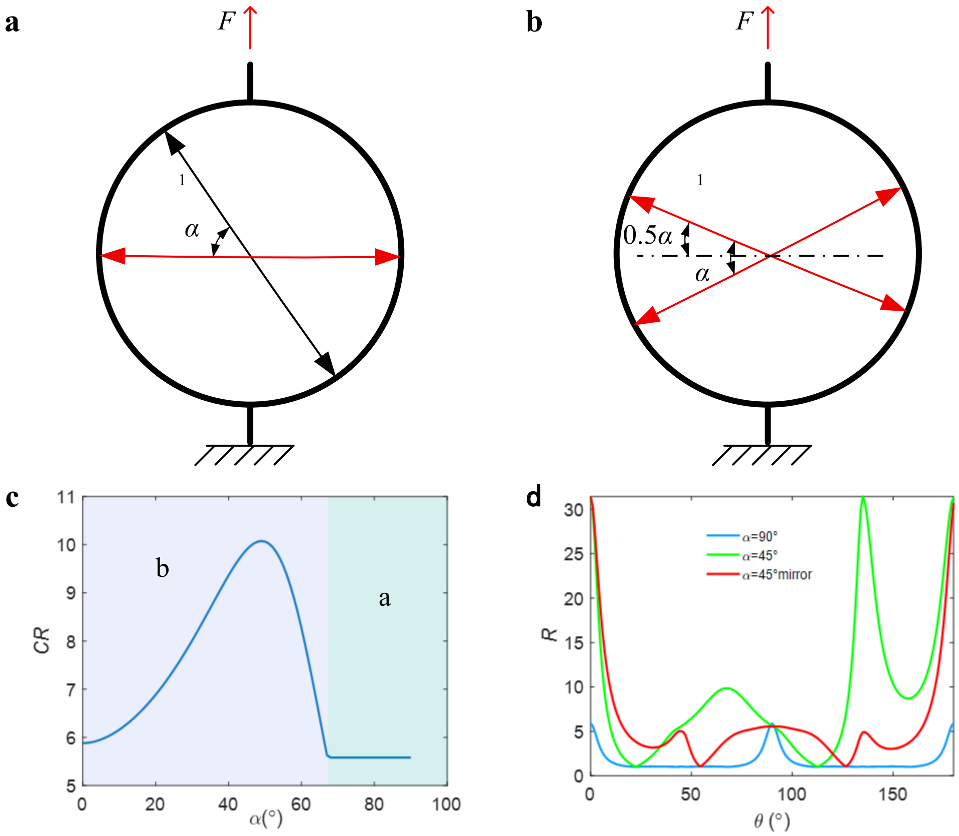

Fig. S13 | Influence of installing angle on the Variable Stiffness. (a,b) Two fulcrum methods corresponds to the maximum tensile stiffness. (c) *CR* changes with *α*. (d) *R* for three cases.

Considering three cases: *α* = 90°, *α* = 45°, *α* = 45° but two adjacent planetary gear units in mirror (Figure 5c in the main manuscript). Then we define the ratios *R* = Larger stiffness/smaller stiffness under different *θ* (The ratio of the larger value to the smaller one between the clockwise and counterclockwise torsional stiffnesses). Based on the analytical formula above, we can obtain *R* of mechanical resonator at different *θ* for the three cases, as shown in **Fig. S13**d. We can see that different *α* and combination method can bring rich variations in *R*, which can introduce different

dynamic characteristics for mechanical resonator. This can meet the needs of different engineering practices.

5. Torsional stiffness of resonators consisting of planetary gear units

Considering the planetary gear unit as a spring, the mechanical resonator can be equivalent to the structure shown in **Fig. S14**. The mechanical resonator is composed of an inner ring (radius R_i), an outer ring (radius R_o) and 8 planetary gear units. In the unloaded state, the radii through the gear unit's inner and outer connection points are defined as R_{o1} and R_{o2}, with β_0 as the initial angle between the unit and R_{o1}, φ_0 as the initial angle between R_{o1} and R_{o2}, and x_0 as the distance from the inner connection point to the perpendicular drawn from R_{o2} to R_{o1}. These parameters (R_i, R_o, β_0, φ_0, x_0) are governed by the geometric relationship described below:

$$\begin{cases}(x_0\tan(\beta_0))^2+(R_i+x_0)^2=R_o{}^2\\ \varphi_0=\arcsin\dfrac{x_0\tan(\beta_0)}{R_o}\end{cases} \tag{29}$$

Based on the structural parameters R_i = 23 mm, R_o = 53 mm, and β_0 = 45°, φ_0 and x_0 can be determined using Eq. (29), with the original length of the variable stiffness unit given as $L = x_0 / \cos\beta_0$. When the inner ring is fixed and a tangential force is applied to the outer ring, the resulting rotation changes the angle between the planetary gear unit and R_{o1} by β, and the angle between R_{o1} and R_{o2} by φ, while the distance from the inner ring connection point to the perpendicular line becomes x. Then the force analysis of the mechanical resonator satisfies the following relation:

$$\begin{cases}(x\tan(\beta_0-\beta))^2+(R_i+x)^2=R_o{}^2\\ L-\dfrac{x}{\cos(\beta_0-\beta)}=\dfrac{F\cos\gamma}{k_r(\theta)}\\ \varphi_0-\varphi=\arcsin\dfrac{x\tan(\beta_0-\beta)}{R_o}\\ \gamma=\dfrac{\pi}{2}-[(\beta_0-\beta)-(\varphi_0-\varphi)]\end{cases} \tag{30}$$

where $k_r(\theta)$ is the equivalent stiffness of the planetary gear unit under different θ. By converting the force into torque $T = F \times R_o$, the relationship between T and φ for different θ can be derived from

Equation (30), as shown in **Fig. S14**. A positive φ represents counterclockwise rotation, and a negative φ represents clockwise rotation.

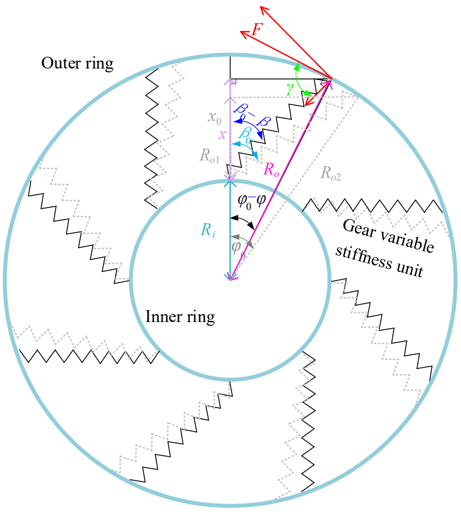

Fig. S14 | Simplified mechanical resonator and its force-deformation analysis

Supplementary **Fig. S15**a shows that at small θ the torque exhibits significant asymmetry between positive and negative directions and the inflection point occurs at φ < 0°. In practical applications, pre-stress in the planetary gear unit introduced by interference fit will shift the inflection point to δ_0 (δ_0< 0°), as shown in **Fig. S15**b.

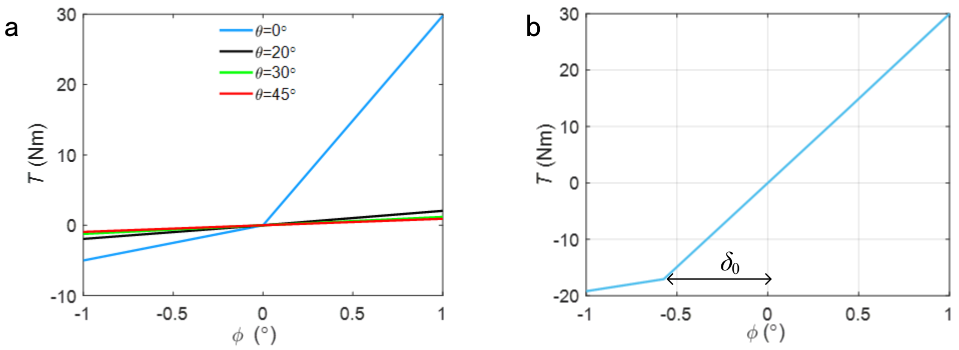

Fig. S15| Relationship between torsional force and torsional deformation of the mechanical resonator for different *θ*. (a)Without pre-stress for different θ. (b) With pre-stress for θ=0°.

As observed in **Fig. S15**a, the torsional force-deformation relationship of the mechanical resonator is nonlinear. This nonlinearity arises from two factors: first, a mild geometric nonlinearity within the same rotational direction (clockwise or counterclockwise), which is weak enough to be approximated as linear; and second, a stronger directional nonlinearity due to the difference in

torsional stiffness between clockwise and counterclockwise directions. When this directional stiffness difference is significant, the nonlinear torsional dynamic behavior of the mechanical resonator becomes pronounced.

6. Tunable nonlinear resonances

6.1 Equations of motion

Based on **Fig. S15**a, the torque T at torsional angle φ=±1° is taken to calculate the torsional stiffness of the vibration absorption structure $K_\theta=T/\varphi$ under different θ. Then, based on the torsional dynamic equation:

$$J\ddot{\varphi}+C\dot{\varphi}+K_\theta\varphi=T,\ K_\theta\varphi=\begin{cases}K_\theta{}^{+}\varphi \text{ for } \varphi\geq\delta_0\\ K_\theta{}^{-}\varphi+(K_\theta{}^{+}-K_\theta{}^{-})\delta_0 \text{ for } \varphi<\delta_0\end{cases} \tag{31}$$

Here T denotes the input torque. Our experimental apparatus has J=0.01 kg·m^2. The δ_0 denotes the pre-deformation angle induced by the assembling process. The interference fit is used between meshing gears to ensure reliable connection, leaving a tiny rotation angle of δ_0≈0.6°. A damping coefficient of C=0.05 Nms/rad is introduced to facilitate convergence. The torsional frequency response can be calculated by time-domain simulation based on Eq.(31). A sweep signal with a frequency range of 1-100 Hz and a duration of 100 seconds is applied as the input. The output response is then processed using the Fast Fourier Transform (FFT) to derive the frequency response. By systematically increasing the amplitude of the sweep signal, the system's response across different input levels is characterized.

6.2 Torsional frequency response of mechanical resonator

The torsional frequency responses for α = 90° and α = 45° at different values of θ are calculated under increasing excitation amplitudes, as summarized in **Fig. S16**.

For α = 90°, three representative cases are examined: θ = 0° (strong nonlinearity), θ = 10° (medium nonlinearity), and θ = 45° (linear). As shown in **Fig. S16**a, under strong nonlinearity, the torsional resonance frequency of the mechanical resonator shifts toward lower frequencies as the excitation amplitude increases, consistent with experimental observations. **Fig. S16**b reveals that this

frequency shift diminishes as nonlinearity weakens. Furthermore, **Fig. S16**c demonstrates that in the absence of nonlinearity, the resonance frequency remains stable even under large excitation amplitudes.

For α = 45°, three configurations are analyzed: θ = 22.5° (linear), θ = 67.5° and θ = 135° (both exhibiting strong nonlinearity). The corresponding torsional frequency responses are shown in **Fig. S16**d-f. Similarly, under strong nonlinear conditions, the resonance frequency shifts toward lower frequencies with increasing excitation amplitude. The above calculation results are also consistent with the experimental results in Figure 5 in the main manuscript.

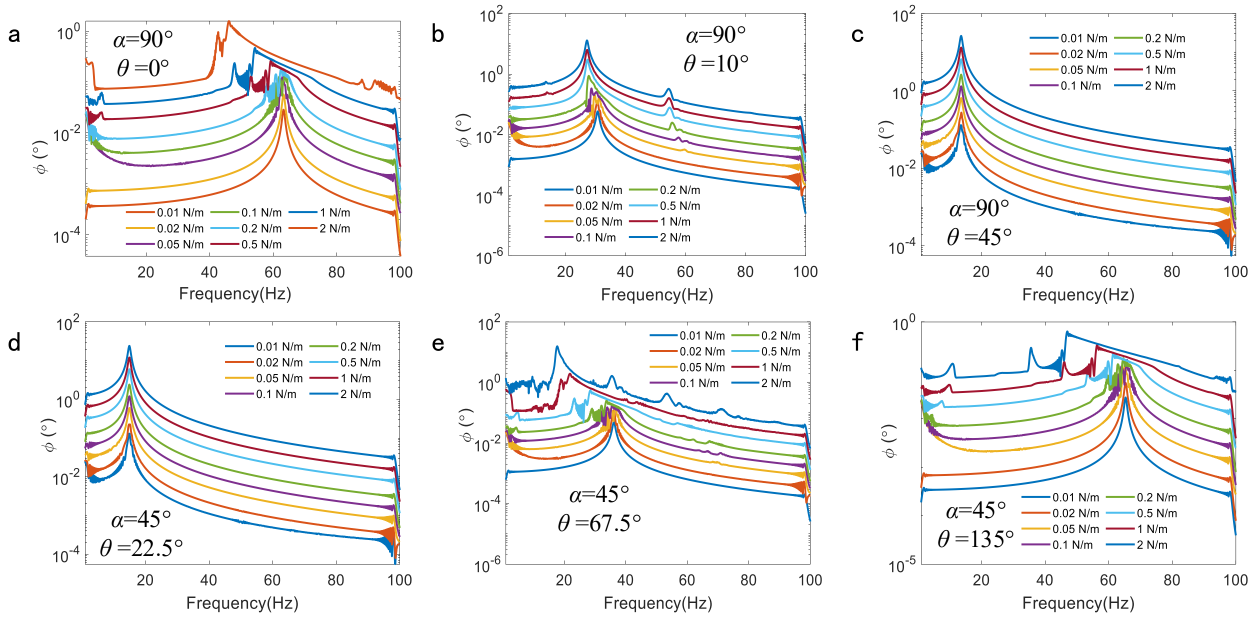

Fig. S16 | Torsional frequency response of the mechanical resonator for differemt *α* and *θ*. (a-c) ***α***=90°. (d-f) ***α***=45°. (a) θ=0°. (b) θ=10°.(c) θ=45°. (d) θ=22.5°.(e) θ=67.5°. (f) θ=135°.

6.3 Harmonic balance method

When α=90° and θ=0°, the relationship between the torque T and the rotation angle φ of the mechanical resonator is shown in **Fig. S17**a. A continuous function is used to fit the piecewise function, assuming $T=k_1\varphi+k_2\varphi^2+k_3\varphi^3$, and the fitted relationship between the T and the φ is shown in **Fig. S17**a. The harmonic balance method is employed to solve the torsional frequency response of the mechanical resonator. Without considering damping, the dynamic equation is as follows:

$$J\ddot{\varphi}+k_1\varphi+k_2\varphi^2+k_3\varphi^3=T_0\sin\omega t \quad (32)$$

Where $T_0\sin\omega t$ is the external torsional excitation. Assuming the solution of the equation is as follows:

$$\varphi = A_0 + A_1 \sin \omega t \tag{33}$$

and substituting Eq.(33) into Eq.(32) while ignoring higher-order harmonics, the following is obtained:

$$\begin{cases} k_1 A_0 + k_2 A_0^2 + k_3 A_0^3 + \frac{1}{2} k_2 A_1^2 + \frac{3}{2} k_3 A_0 A_1^2 = 0 \\ (-m\omega^2 + k_1) A_1 + 2k_2 A_0 A_1 + 3k_2 A_0^2 A_1 + \frac{3}{4} k_3 A_1^3 - T_0 = 0 \end{cases} \tag{34}$$

Based on Eq.(34), the values of A_0 and A_1 characterizing the torsional frequency response of the mechanical resonator can be determined. Applying this approach, the torsional frequency response under an excitation amplitude of T_0 = 2 Nm is obtained, as illustrated in **Fig. S17**b. Owing to nonlinear effects, the solutions for A_0 and A_1 exhibit bifurcation within the frequency range of 1–54 Hz, yielding multiple solution branches, among which certain branches are unstable. Furthermore, the frequency response derived via the harmonic balance method reveals a resonance shift toward lower frequencies, a phenomenon consistent with both experimental observations and time-domain simulation results.

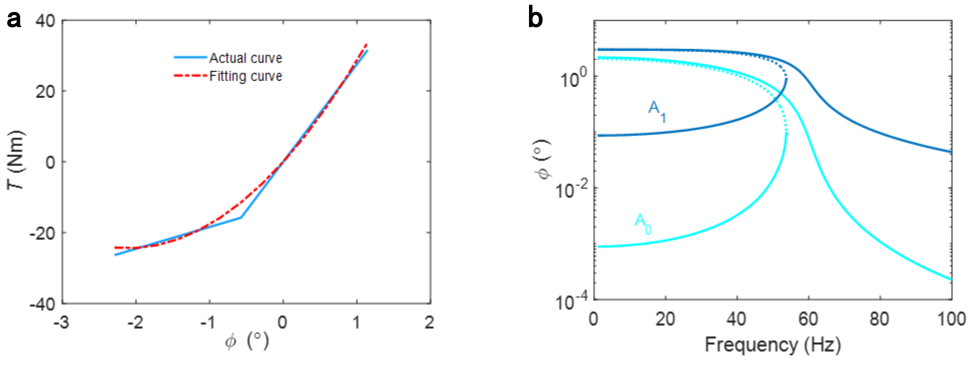

Fig. S17 | Harmonic balance method. (a) Approximate a piecewise function with a continuous function. (b) The A0 and A1 components of the torsional frequency response under T = 2 Nm via the harmonic balance method.

[1] Chen J C .Deflection Equations and Formulas of Curved Beams and their Applications (Particularly of Thin Curved Beams with Circular Axial Line and Constant Cross Section)[J]. 1957.
